# Blood–Brain Barrier Transporters: Opportunities for Therapeutic Development in Ischemic Stroke

**DOI:** 10.3390/ijms23031898

**Published:** 2022-02-08

**Authors:** Kelsy L. Nilles, Erica I. Williams, Robert D. Betterton, Thomas P. Davis, Patrick T. Ronaldson

**Affiliations:** Department of Pharmacology, College of Medicine, University of Arizona, Tucson, AZ 85724-5050, USA; knilles@email.arizona.edu (K.L.N.); eiwilliams@email.arizona.edu (E.I.W.); rdbetter@email.arizona.edu (R.D.B.); davistp@email.arizona.edu (T.P.D.)

**Keywords:** blood–brain barrier, endothelial cell, ischemic stroke, neuroprotection, organic anion transporting polypeptides, organic cation transporters, statins, transporters

## Abstract

Globally, stroke is a leading cause of death and long-term disability. Over the past decades, several efforts have attempted to discover new drugs or repurpose existing therapeutics to promote post-stroke neurological recovery. Preclinical stroke studies have reported successes in identifying novel neuroprotective agents; however, none of these compounds have advanced beyond a phase III clinical trial. One reason for these failures is the lack of consideration of blood–brain barrier (BBB) transport mechanisms that can enable these drugs to achieve efficacious concentrations in ischemic brain tissue. Despite the knowledge that drugs with neuroprotective properties (i.e., statins, memantine, metformin) are substrates for endogenous BBB transporters, preclinical stroke research has not extensively studied the role of transporters in central nervous system (CNS) drug delivery. Here, we review current knowledge on specific BBB uptake transporters (i.e., organic anion transporting polypeptides (OATPs in humans; Oatps in rodents); organic cation transporters (OCTs in humans; Octs in rodents) that can be targeted for improved neuroprotective drug delivery. Additionally, we provide state-of-the-art perspectives on how transporter pharmacology can be integrated into preclinical stroke research. Specifically, we discuss the utility of in vivo stroke models to transporter studies and considerations (i.e., species selection, co-morbid conditions) that will optimize the translational success of stroke pharmacotherapeutic experiments.

## 1. Introduction

According to 2019 epidemiological data, there were 12.2 million cases of stroke globally, which resulted in 6.55 million deaths [1]. Ischemic strokes are the most common type and comprise approximately 87% of all strokes [2]. Ischemic stroke pathophysiology involves reduced supply of glucose and oxygen to an affected brain region resulting in an occluded blood vessel, a process that leads to an irreversibly damaged infarction core as well as potentially salvageable surrounding tissue known as the penumbra [3]. Treatment of the ischemic core is impossible due to rapid progression of necrosis; however, the penumbra is a primary target for therapeutic intervention due to slower cell degradation [4,5,6]. To date, approved treatments for ischemic stroke include thrombolysis (i.e., recombinant tissue plasminogen activator (r-tPA; alteplase)) and surgical interventions (i.e., endovascular thrombectomy (EVT)). Treatment with r-tPA is limited by its narrow therapeutic window (i.e., 4.5 h) and/or risk of bleeding complications [4]. In fact, the National Institute of Neurological Disorders and Stroke (NINDS) reported that the clinical risk of symptomatic intracerebral hemorrhage (SICH) for patients that received r-tPA was 6.4% as compared to 0.6% for patients that were administered placebo [7]. Risk factors for SICH post-thrombolytic therapy include stroke severity, elevated serum glucose concentrations, time to treatment, elevated systolic blood pressure, low platelets, and advanced age [8]. Mechanical EVT has provided considerable benefits to stroke patients such as improved cerebral reperfusion [9,10]; however, severe disability remains a clinically significant problem in patients treated via EVT [10,11,12]. It is important to consider that r-tPA and EVT, the only two measures available for immediate treatment of acute ischemic stroke, involve recanalization (i.e., reperfusion) of ischemic brain tissue, a process that can dramatically exacerbate neuronal damage. Central nervous system (CNS) injury following recanalization ranges in severity from ischemic core enlargement to the development of edema or fatal hemorrhaging, a critical component of ischemic/reperfusion (I/R) injury [6]. Pathological processes associated with I/R injury include enhanced blood-brain barrier (BBB) permeability and leakage, activation of cell death processes (i.e., apoptosis, autophagy, necrosis), neuroinflammation, and oxidative stress [13,14,15,16,17].

The common utilization of reperfusion therapies for treatment of acute ischemic stroke emphasizes a critical need for strategies that can protect neuronal tissue from further damage and/or promote neuronal repair following I/R injury. Several currently marketed drugs and experimental therapeutics have been reported to exert neuroprotective effects in preclinical stroke studies. In fact, approximately 95% of studies that have been reported in the scientific literature between 1990 and 2018 indicated positive neuroprotective outcomes in vivo; however, none of these studies have resulted in advancement of a therapeutic beyond a Phase III clinical trial [10]. There are several reasons for mismatches between successful preclinical experiments and failed clinical trials including: (i) lack of rigorous behavioral studies to assess functional neurological outcomes; (ii) therapeutic effects were attained at high doses in animal models that cannot be safely administered to human subjects; (iii) in vivo studies are often conducted in young animals that do not reflect challenges in treatment of acute/subacute/chronic ischemic stroke that are apparent in older human subjects where stroke is more prevalent; and (iv) in vivo studies often do not incorporate comorbid conditions (i.e., diabetes mellitus, tobacco smoking, hypertension, atrial fibrillation) that are common in individuals that experience acute ischemic stroke [10,18]. An additional consideration is that studies examining neuroprotection and/or neural repair in experimental stroke have not evaluated specific biological mechanisms (i.e., transporters) that can be targeted to enable drugs to efficiently permeate the BBB. This critical consideration is highlighted by the clinical failure of the antioxidant drug disufenton sodium (i.e., NXY-059, Cerovive^®^) that was developed as a stroke therapeutic. As noted in our recent review [6], BBB penetration of disufenton sodium, a polar molecule with limited lipophilicity, was not rigorously assessed during preclinical development. If detailed BBB transport studies had been conducted, it would have been revealed that disufenton sodium was a transport substrate for organic anion transporters (OATs in humans; Oats in rodents) [19]. At the BBB, the predominant OAT/Oat isoform is OAT3/Oat3, which functions to transport its substrates in the brain-to-blood direction [20,21,22]. This property is likely to have contributed to limited CNS penetration and reduced clinical effectiveness of disufenton sodium. 

The experience with disufenton sodium emphasizes that drug discovery and/or development for treatment of ischemic stroke requires the study of specific mechanisms that directly facilitate CNS drug delivery. Indeed, transport of a therapeutic agent at the cerebral microvasculature can involve passive diffusion (i.e., transcellular or paracellular), carrier-mediated transport, receptor-mediated transcytosis, or absorptive transcytosis [20,23]. The BBB possesses several endogenous transport proteins that allow for selective uptake of required nutrients or restrict brain delivery of potentially toxic xenobiotics [6,17,24]. To date, the majority of published studies on BBB transporters in ischemic stroke models have focused on glucose or ion transport mechanisms [25,26,27,28]. With respect to small-molecule therapeutic agents, there are a few reports on changes in brain-to-blood drug transport by efflux transporters in the setting of experimental stroke [29,30,31] and no reports on endogenous BBB uptake transporters for drugs. This is a significant knowledge gap because transporters that can move substrates in the blood-to-brain direction represent a translational opportunity to optimize drug concentrations in the ischemic brain and, subsequently, improve pharmacotherapy of ischemic stroke. In this review, we highlight those endogenous transport mechanisms that have the potential to be targeted for optimization of CNS delivery of small molecule drugs. We also emphasize practical considerations (i.e., differences between preclinical animal species, stroke model selection, inclusion of comorbid conditions into transport studies) that must be considered in drug delivery studies utilizing animal models of ischemic stroke. This knowledge is critical to the discovery of new chemical entities and/or repurposing of existing drugs for improvement of functional neurological outcomes following ischemic stroke. Indeed, several preclinical stroke studies have explored other technologies for CNS delivery of drugs including nanoparticles [32,33,34], liposomes [35,36], dendrimers [37], or therapeutic antibodies targeting the transferrin receptor for receptor-mediated transcytosis [38]. While these approaches have shown varying degrees of potential for clinical translation, they do not utilize putative membrane transporters that are functionally expressed at the BBB and, therefore, will not be discussed in this review.

## 2. The Blood–Brain Barrier and the Neurovascular Unit

**Figure 1:**
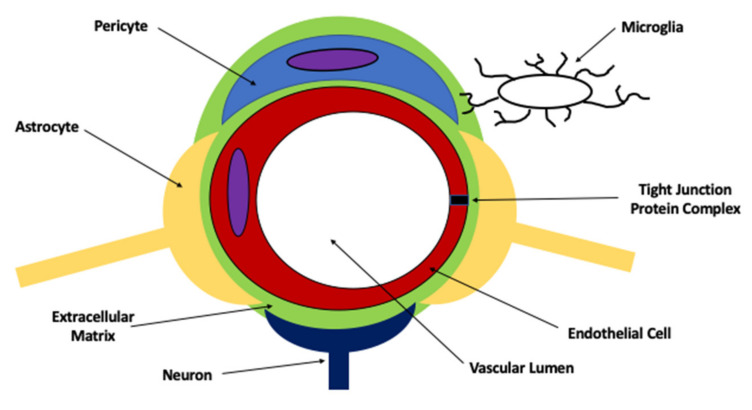
Anatomy of the neurovascular unit.

The BBB exists at the level of the brain microvascular endothelium. A central concept in BBB physiology is that endothelial cells cannot form barrier characteristics without coordinated communication networks with other CNS cell types (i.e., astrocytes, microglia, pericytes, neurons) and the extracellular matrix (Figure 1) [17,39]. This relationship implies the existence of a “neurovascular unit (NVU)”, a concept that was formally defined by the NINDS Stroke Progress Review Group in 2001 [40]. By emphasizing the symbiotic nature of cell-cell interactions between endothelial cells, glial cells (i.e., astrocytes, microglia), pericytes, and neurons as well as contributions from extracellular matrix constituent enzymes and proteins, the concept of the NVU caused a dramatic ideological shift in our understanding of neurological diseases [40]. Instead of acting as independent entities, it is now believed that CNS cellular constituents interact directly with cerebral microvasculature to enable endothelial cells to develop distinct barrier properties and to allow for dynamic responses to pathological stressors. In the context of ischemic stroke, BBB dysfunction is now understood to be an early pathological event that occurs prior to neuronal injury and death [41,42]. Pathophysiological mechanisms associated with BBB dysfunction in stroke have been recently reviewed by our laboratory [17] and will not be discussed further in this review. 

### 2.1. Blood–Brain Barrier Transport Mechanisms for Small Molecules

At the BBB, transporters are a critical mechanism that selectively permits brain uptake of circulating solutes while restricting the permeability of others [6]. In the context of ischemic stroke, the role of transporters at the brain microvascular endothelium must be understood in conjunction with knowledge that the BBB is disrupted in the first few hours following an ischemic insult [17]. This pathological event involves altered localization and expression of individual tight junction constituent proteins as well as reorganization of oligomeric tight junction protein assemblies. Indeed, decreased expression of tight junction constituent proteins can occur in response to ischemia and contribute to BBB dysfunction. For example, transmembrane tight junction proteins (e.g., claudin-3, occludin) were shown to be downregulated following middle cerebral artery occlusion (MCAO) in mice, an effect that led to the enlargement of cerebral infarction and the development of vasogenic edema [43]. Additionally, collapse of occludin oligomeric assemblies and movement of occludin away from the tight junction were observed in cerebral microvessels in rats subjected to hypoxia/reoxygenation stress, a component of stroke pathogenesis [44]. The result of altered occludin oligomerization is increased paracellular “leak” to a small molecule tracer (i.e., [^14^C] sucrose) between adjacent endothelial cells in the region of the hypoxic insult [44]. Tight junctions play a critical role in regulation of water and solute movement between endothelial cells (i.e., paracellular diffusion) via the formation of a physical barrier [17,45,46]. Protein constituents of BBB tight junctions include, but are not limited to, transmembrane proteins such as the claudins and occludin as well as intracellular accessory proteins such as zonula occluden (ZO) proteins. Claudins, in particular claudin-5, are central to the formation of the physiological “seal” of the tight junction and thereby restrict paracellular diffusion [47,48,49]. The prominent role of claudin-5 at the BBB is emphasized by the fact that claudin-5 null mice die immediately after birth [50]. The mechanism for postnatal mortality in mice following global claudin-5 knockout involves enhanced cerebral microvascular paracellular permeability, which was reflected by an increase in blood-to-brain diffusion of small molecules (i.e., gadolinium-diethylene triamine-*N,N,N’,N”,N”*-pentaacetic acid, Hoechst 33258), as well as altered water homeostasis [50]. Additionally, occludin is a critical regulator of BBB functional integrity, predominantly via its ability to assemble into dimers and higher-order oligomers [44,51,52]. Transmembrane tight junction proteins are directly linked to the endothelial cell cytoskeleton by interactions with ZO proteins, which are members of the membrane-associated guanylate kinase-like (MAGUK) protein family. Several reports in the scientific literature have shown that altered ZO-1 expression and/or localization is associated with increased BBB permeability [53,54,55], suggesting that ZO-1 is critical to the stability and function of the tight junction. Interestingly, transport proteins for small molecules can overcome changes in tight junction integrity and subsequent paracellular “leak” at the BBB. This enables transporters to remain as primary determinants of CNS drug delivery despite dysfunction at the level of the tight junction. Our laboratory has demonstrated this very concept using an in vivo rodent model of pain/inflammation where pathophysiological stress at the BBB is induced by injection of λ-carrageenan into the plantar surface of the right hind paw [56]. We have shown that tight junction protein complex integrity and paracellular permeability to a small molecular weight tracer (i.e., sucrose) are both enhanced following λ-carrageenan administration [51,57,58,59]; however, we also demonstrated that brain exposure to morphine (i.e., a transport substrate for the critical efflux transporter P-glycoprotein (P-gp)) in saline-treated animals and in λ-carrageenan-injected animals could only be increased in the presence of the competitive P-gp inhibitor cyclosporine A [56]. This novel result implies that transcellular transport remains a principal determinant of blood-to-brain delivery of morphine despite the opening of a non-selective passive diffusion route between adjacent BBB endothelial cells.

Paracellular permeability at the BBB is also regulated by adherens junctions. These specialized cell–cell interactions involve cadherins as well as other associated proteins that are directly linked to the actin cytoskeleton [6]. Cadherins activate PI3K signaling, a mechanism that is critical for maintenance of endothelial homeostasis and organization in response to angiogenesis [60]. Brain microvascular endothelial cells are known to express high levels of cadherin-10 protein relative to VE-cadherin [61]. In contrast, CNS vascular structures that lack barrier properties (i.e., circumventricular organs, choroid plexus) primarily express VE-cadherin [61]. Cadherin proteins are known to physically associate with catenins, a process that is absolutely required to ensure the optimal function of cadherin at the adherens junction. In mammalian endothelial cells, four catenin isoforms (designated α, β, χ, and p120) have been identified. The most critical interaction for the formation of adherens junctions at the BBB involves the binding of cadherin and α-catenin to the actin cytoskeletal via β-catenin [62]. β-catenin also interacts with Wnt signaling molecules in brain microvascular endothelial cells, an effect that is required for the development of the BBB phenotype [63,64]. Additionally, the heparan sulfate proteoglycan agrin has been shown to promote the proper localization of cadherins and catenins at brain endothelial cellular junctions [65]. Under pathological conditions, increased expression of cadherins and catenins at plasma membrane sites between opposing endothelial cells is associated with BBB repair [66]. This concept has been demonstrated for VE-cadherin, which is upregulated in vascular endothelial cells following experimental ischemic stroke in mice [67].

**Figure 2:**
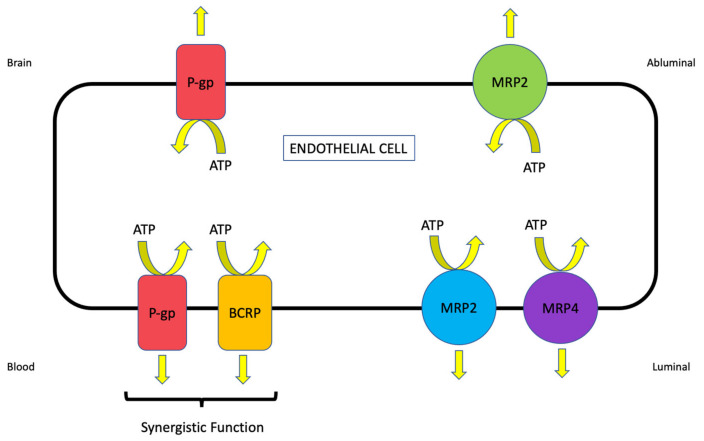
Localization of ATP-Binding Cassette (ABC) transporters at the Blood-Brain Barrier (BBB). ABC efflux transporters that are known to play a critical role in central nervous system (CNS) drug disposition are shown. All of these transporters function as primary active transporters and utilize ATP as an energy source to move drug molecules against their concentration gradient. Current knowledge in the field implies that P-glycoprotein (P-gp) and Breast Cancer Resistance Protein (BCRP) function in synergy to restrict blood-to-brain transport of therapeutics [6,69].

BBB transporters for small molecules belong to one of two protein superfamilies: ATP-binding cassette (ABC) and solute carrier (SLC) transporters [6]. ABC transporters function as primary active transporters that hydrolyze ATP to drive the movement of drugs and metabolites against their concentration gradient. In the context of stroke, these transporters constitute an obstacle to effective CNS drug uptake by limiting access to brain tissue. Indeed, brain microvascular endothelial cells have evolved to have an increased number of mitochondria to support the production of the biological energy required to drive drug transport across the BBB [68]. Members of the ABC superfamily that are functionally expressed at the mammalian BBB include P-gp, multidrug resistance proteins (MRPs in humans; Mrps in rodents), and breast cancer resistance protein (BCRP in humans, Bcrp in rodents) (Figure 2) [6,69]. In contrast, blood-to-brain transport of therapeutic agents involves members of the SLC family that, in humans, is comprised of 395 members that are organized into 52 families [70]. Transport mechanisms for SLC family members include facilitated diffusion (i.e., transport in the direction of the substrate concentration gradient) or secondary/tertiary active transport (i.e., transport is driven by transmembrane ion/solute gradients established by other primary or secondary active transporters) [5]. Regardless of the type of transport mechanism, SLC members exhibit different specificities and affinities for structurally diverse substrates [5,24]. Of particular relevance to the treatment of ischemic stroke, drugs that are known transport substrates for SLC transporters include 3-hydroxy-3-methylglutaryl coenzyme A (HMG-CoA) reductase inhibitors (i.e., statins) and the N-methyl-D-aspartate receptor (NMDAR) antagonist memantine [6]. Current knowledge on transporter localization at the BBB across multiple preclinical species is presented in Table 1. Representative centrally active substrates for ABC and SLC transporters that are expressed at the BBB are presented in Table 2.

### 2.2. Role of Efflux Transporters in Drug Disposition to the Ischemic Brain

Perhaps the most critical efflux transporter with respect to CNS drug penetration is P-gp, a 170 kDa integral transmembrane protein that is highly expressed in brain microvascular endothelial cells. P-gp is encoded by the multidrug resistance (*MDR*) gene that has two isoforms in humans (i.e., *MDR1* and *MDR3*) and three isoforms in rodents (i.e., *mdr1a, mdr1b, mdr2*). Across several species, P-gp has been primarily localized to the luminal plasma membrane of cerebral endothelial cells [75,82,83,84,85,87]. Additionally, P-gp has been detected at the abluminal plasma membrane of endothelial cells from human and rat brain tissue, which suggests that this transporter may play a critical role in maintaining brain concentrations of specific substrates required for CNS homeostasis [84]. Transport substrates for P-gp are typically small molecules that range in molecular weight from 250 Da to over 1200 Da [101]. Examples include, but are not limited to, antibiotics, anti-cancer agents, antihistamines, antiretrovirals, β-blockers, calcium channel blockers, cardiac glycosides, ionophores, immunosuppressants, opioid analgesics, statins, and steroids [20].

Previous studies have demonstrated that P-gp functional expression is altered at the BBB following an ischemic insult. Using the transient middle cerebral artery occlusion (MCAO) model (30 and 90 min occlusion), Spudich and colleagues demonstrated that P-gp protein expression is elevated in CD31-positive microvascular endothelial cells as early as 3 h post-focal cerebral ischemia [29]. This elevation persisted up to 24 h following MCAO in both cortical tissue and in the striatum and returned to baseline protein expression levels by 72 h [29]. P-gp expression has also been reported to increase at the BBB following 90 min MCAO as well as in cultured rat brain endothelial cells subjected to hypoxia and low glucose conditions [31]. More recently, I/R injury was shown to enhance cerebral expression of multiple ABC transporters at the mRNA level including P-gp and various Mrp isoforms (i.e., Mrp1, Mrp2, Mrp4, Mrp5) [102]. A significant limitation of the study by Zhu and colleagues is that transporter gene expression was not evaluated in specific cellular compartments of the NVU including endothelial cells [102]. Therefore, these data cannot be used to make inferences regarding the effects of I/R injury on ABC transporter mRNA expression in cerebral microvasculature. Nonetheless, altered functional expression of P-gp can have profound implications for CNS delivery of therapeutics relevant to pharmacotherapy of ischemic stroke. Indeed, several drugs with neuroprotective properties have been shown to be P-gp transport substrates. For example, P-gp has been shown to play a role in CNS disposition of tanshinone IIA, a component of *Salvia miltiorrhiza* Bunge that has been proposed as a stroke therapeutic [103]. More recently, studies in human endothelial cells have identified commonly prescribed statin drugs such as atorvastatin and rosuvastatin as P-gp transport substrates [100]. Such observations may imply that pharmacological inhibition of P-gp can be an effective strategy to increase drug delivery to ischemic brain tissue. Proof-of-concept studies using the competitive inhibitor tariquidar have shown that blockade of P-gp transport activity can increase the brain-to-blood concentration ratio of two drugs with neuroprotective effects (i.e., FK506 and rifampicin) [29]; however, this strategy is limited by the ubiquitous expression profile of P-gp [24]. Indeed, P-gp is expressed in multiple barrier epithelial cells including colon, small intestine, renal proximal tubules, and bile canaliculi. As such, inhibition of P-gp at the BBB using pharmacological antagonists will also block P-gp function in other tissues, an effect that can increase the disposition of drugs throughout the body and enhance the probability of off-target effects and/or dose-limiting toxicities [104,105]. Furthermore, pharmacokinetic modeling of clinical P-gp inhibition has predicted that the maximal increase in CNS drug disposition that could be achieved by blocking its activity at the BBB is no more than two-fold [106]. Therefore, the magnitude of increase in a P-gp substrate drug achieved in brain tissue following inhibition of this efflux transporter may not be sufficient to provide therapeutic effectiveness in the setting of ischemic stroke.

P-gp is known to function in synergy with BCRP/Bcrp, a 72 kDa protein that is encoded by the *ABCG2* gene [107]. It is commonly referred to as “half-transporter” and requires the formation of homodimers, tetramers, and duodecamers [108]. Similar to P-gp, BCRP/Bcrp is primarily localized to the luminal plasma membrane in brain microvessel endothelial cells [88,89,90,109]. More recently, Billington and colleagues utilized quantitative targeted proteomics and reported that the relative abundance of BCRP was higher at the human BBB as compared to P-gp [110]. In terms of substrate profile, BCRP has considerable overlap with P-gp and also transports sulfoconjugated organic anions, amphiphilic, and hydrophobic drugs [111]. Endogenous substances such as folic acid, glutathione (GSH) metabolites, and hormones are also established BCRP/Bcrp substrates [112,113,114,115]. Functional expression of BCRP/Bcrp has been shown to be altered in response to pathophysiological conditions relevant to ischemic stroke. Specifically, studies of a co-culture model comprised of bovine brain endothelial cells and rat astrocytes that was subjected to oxygen/glucose deprivation (OGD) conditions demonstrated a reduction in endothelial *ABCG2* mRNA following 4 h OGD [116]. Interestingly, *ABCG2* transcript expression returned to baseline levels after reoxygenation for 24 h or 48 h [116]. It is critical that this finding be validated using in vivo stroke models (i.e., MCAO), particularly due to the fact that BCRP has been shown to play a role in CNS disposition of stroke therapeutics including statins [100].

### 2.3. Role of Uptake Transporters in Drug Disposition to the Ischemic Brain

**Figure 3:**
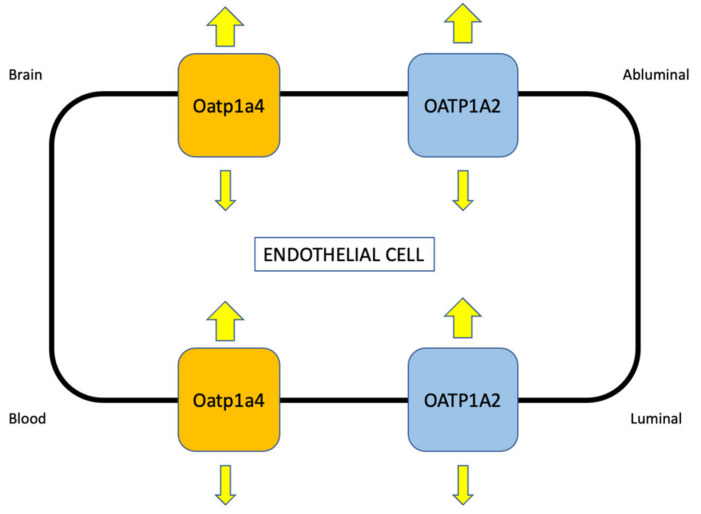
Localization of drug-transporting organic anion transporting polypeptides (OATPs/Oatps) at the Blood-Brain Barrier (BBB). The rodent Oatp isoform Oatp1a4 and its human orthologue OATP1A2 are expressed at the luminal and abluminal plasma membrane of brain microvascular endothelial cells [71,76,77,78]. The driving force for these transporters is the transmembrane concentration gradient. Therefore, they will primarily facilitate blood-to-brain uptake of transport substrates when a therapeutic is administered via the systemic circulation.

Perhaps a more effective strategy for optimization of neuroprotective drug delivery to the ischemic brain is to target uptake transporters at the BBB. Over the past several years, our laboratory has studied functional expression of SLC transporters such as organic anion transporting polypeptides (OATPs in humans; Oatps in rodents) at the cerebral microvasculature and the role of these transporters in CNS drug disposition (Figure 3). We have focused our research on Oatp1a4, the primary drug transporting Oatp isoform expressed at the rat BBB [117,118,119,120,121,122]. In situ hybridization histochemistry, immunofluorescence microscopy, and Western blot analysis of isolated brain microvessels have confirmed localization and/or protein expression of Oatp1a4 [72,78,79,117]. Of particular note, double-labeling experiments with antibodies targeted against Oatp1a4 and glial fibrillary acidic protein (GFAP), an astrocyte marker, demonstrated Oatp1a4 localization at both the luminal and abluminal plasma membrane of capillary endothelial cells [73]. A human orthologue of Oatp1a4 has been identified in brain microvessels from human cerebral cortical tissue and is designated OATP1A2 [76]. OATP1A2 was shown to be one of the most abundant BBB transporters in brain capillaries derived from frontal cortical tissue donated by healthy individuals [123]. In terms of the transport mechanism, OATP1A2 and Oatp1a4 are believed to function primarily as facilitative transporters where the driving force across a biological membrane is dictated by the transmembrane concentration gradient. Such a mechanism suggests that the substrate drug is introduced into the endothelial cell cytoplasm and then attains a critical threshold, which causes a change in direction of the transmembrane concentration gradient and enables the OATP/Oatp transporter to move its substrates in the opposite direction (Figure 3). The process becomes readily apparent under conditions where OATP/Oatp functional expression is increased, such as in response to pathological stressors including pain and/or cerebral hypoxia [117,119] or following activation of molecular signaling pathways that regulate OATP/Oatp expression and activity [120]. Indeed, upregulation of OATP/Oatp transport results in a statistically significant increase in the influx constant (K_in_) and, simultaneously, a small but significant enhancement in the efflux constant (k_out_) [117,119,120].

The relevance of OATPs/Oatps in the treatment of ischemic stroke is directly related to their ability to facilitate blood-to-brain uptake of therapeutics with neuroprotective properties. We have identified that OATPs/Oatps are required to enable CNS penetration of statins, a mechanism that enables these drugs to exert beneficial effects in the setting of ischemic stroke. Much of the experimental evidence for BBB statin transport is derived from in vivo analyses. Using a rodent model of global cerebral hypoxia/reoxygenation stress, Thompson and colleagues reported CNS delivery of the commonly prescribed drug atorvastatin by an Oatp-mediated mechanism [119]. Abdullahi and colleagues expanded upon these observations and provided evidence for brain uptake of atorvastatin and pravastatin via Oatp1a4 [120]. Both of these studies corroborate data from Oatp1a4(−/−) mice where brain accumulation of pitavastatin and rosuvastatin was reduced relative to wild-type controls [71]. It is well known that species differences in drug transport properties exist between rodent models and human cells and/or tissues. This is certainly the case for OATPs/Oatps where distinct differences in transport properties for anti-migraine triptan drugs have been reported [124]. Specifically, Liu and colleagues showed no difference in uptake transport for multiple triptans (i.e., almotriptan, naratriptan, sumatriptan, rizatriptan, zolmitriptan) between Oatp1a4(−/−) mice and wild-type mice, which implies that these drugs are not Oatp1a4 substrates [124]. In contrast, zolmitriptan was identified as an OATP1A2 transport substrate, thereby showing a clear difference in triptan transport properties between rodents and humans [124]. Studies in Madin-Darby canine kidney (MDCK) II cells transfected with OATP1A2 [124] and in human umbilical vein endothelial cells that endogenously express OATP1A2 [100] showed that various statin drugs (i.e., atorvastatin, pravastatin, and/or rosuvastatin) are substrates for this human OATP isoform. These results emphasize the translational utility of using preclinical rodent models to study statin transport properties at the BBB, particularly in the setting of ischemic stroke. Other OATP/Oatp transport substrates that may be relevant to ischemic stroke treatment include opioid analgesic peptides. Using brain microvascular endothelial cells differentiated from human induced pluripotent stem cells (iPSC-BMECs), Albekairi and colleagues showed that cellular uptake of biphalin, a potent mu-opioid receptor, and delta-opioid receptor agonist, was reduced in the presence of estrone-3-sulfate, a known OATP inhibitor [125]. OATP1A2 protein expression was confirmed in the iPSC-BMEC cell culture model using fluorescent immunocytochemistry [125]. Additionally, biphalin uptake was increased in iPSC-BMECs subjected to oxygen-glucose deprivation (OGD) conditions, an in vitro experimental paradigm relevant to ischemic stroke [125]. Opioid receptor agonists have been previously shown to exert neuroprotective effects in preclinical stroke models [126,127,128,129,130,131]. Therefore, targeting OATP-mediated transport at the BBB represents a mechanism that can facilitate the delivery of these potentially efficacious neuroprotective drugs to the ischemic brain.

In addition to OATPs/Oatps, organic cation transporters (OCTs in humans, Octs in rodents) can be targeted for blood-to-brain delivery of stroke therapeutics. Previous studies have reported Oct mRNA in cultured brain endothelial cells or brain microvessels [132,133]. Expression of OCT1/Oct1, OCT2/Oct2, and OCT3/Oct3 has been detected at the protein level in an immortalized mouse brain endothelial cell line (bEND3) and in a human brain microvessel endothelial cell line (hCMEC/d3) [80]. These results are consistent with a previous study by Lin and colleagues that showed increased luminal expression of Oct1 and Oct2 in cultured rat brain endothelial cells [81]. Using fluorescent confocal microscopy, our laboratory has detected Oct1 in brain microvessels isolated from female Sprague-Dawley rats [5,134]. Similar to OATP isoforms, global proteomic analysis of human brain microvessels revealed detectable quantities of OCT1 and OCT3 protein [123]. In contrast, quantitative targeted proteomics reported that both OCT1 or OCT2 were below the limit of detection at the BBB in hippocampal Brodmann Areas 17 and 39 [110]. When compared to the study by Al-Majdoub and colleagues, these data imply regional differences in OCT localization/expression at the BBB. Therefore, it is essential that such results be confirmed by detailed molecular studies and functional assays.

**Figure 4:**
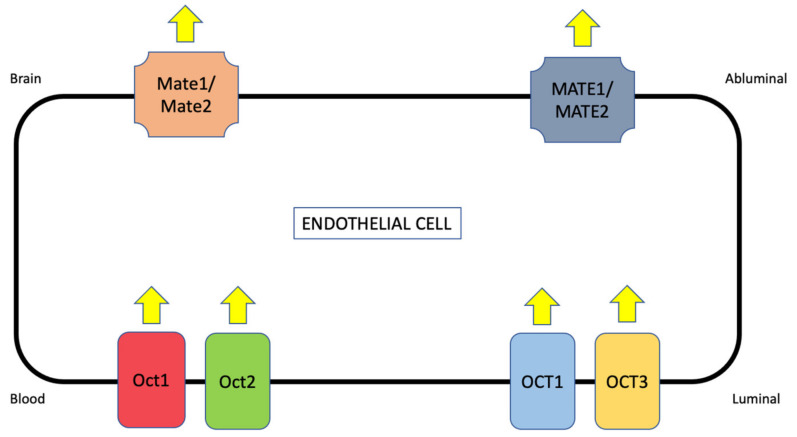
Proposed localization of organic cation transporters (OCTs/Octs) and multidrug and toxin extruders (MATEs/Mates) at the Blood-Brain Barrier (BBB). Due to their polarized nature, OCT/Oct isoforms are believed to be localized to the luminal plasma membrane in brain microvascular endothelial cells while MATE/Mate transporters are localized to the abluminal plasma membrane. These SLC transporters function as secondary active transporters that are coupled to a proton gradient to drive substrate transport in the blood-to-brain direction.

Translocation of cationic substances across biological membranes in polarized cells involves a two-step process where OCTs/Octs are involved in initial cellular uptake. An excellent example is organic cation secretion in the renal proximal tubule, which requires transport synergy between OCT2 and the multidrug and toxin extrusion transporter 1 (MATE1) [99]. MATE1 functions to ensure the extrusion of cationic substrates into the urinary filtrate so that they can be efficiently excreted. At the BBB, current evidence suggests that OCTs/Octs are preferentially expressed at the luminal plasma membrane of endothelial cells (Figure 4) [134]. Functional expression of Octs has been demonstrated in brain microvessel endothelial cells as shown by OCT1/Oct1 and OCT2/Oct2-mediated uptake of N-methyl-4-phenyl-1,2,3,6-tetrahydropyridine (MPTP) [81]. The fact that MPTP can completely cross the BBB suggests that additional transporters such as MATEs are expressed to ensure effective CNS delivery of cationic drugs. Recently, protein expression of MATE1 and MATE2 was observed in hCMEC/d3 cells [80]. While the exact localization has not been confirmed in mouse brain endothelial cells, Mate1 and Mate2 protein expression was detected in cerebral microvasculature from C57BL6/129 mice [80] and Mate1 gene and protein expression was detected in brain capillaries from male ddY mice [135]. More recently, Mate1 mRNA was shown to be expressed at the BBB in Swiss, FVB and C57BL6/JRj mice [136]. Of particular significance, MATE1 and MATE2 protein was detected by Western blot analysis in the human frontal cortex, caudate nucleus, and putamen [80]. These results are consistent with immunofluorescence staining of human brain microvessels, which confirmed MATE1 expression at the BBB [137]. In contrast, Chaves and colleagues failed to detect MATE isoforms in brain microvessels derived from human glioma tissue [136]. It is possible that pathophysiological factors associated with tumorgenesis or cancer pharmacotherapy downregulated MATEs in these tissue samples, thereby accounting for these discordant results. The variability in MATE/Mate expression data represented by these studies does emphasize the need to clarify the involvement of MATE/Mate isoforms as transporters that function in concert with OCTs/Octs to deliver cationic drugs to the brain.

BBB transport of memantine represents a proof-of-concept example of the therapeutic potential of targeting OCTs in the setting of ischemic stroke. Pharmacodynamically, memantine functions as an antagonist of N-methyl-D-aspartate receptors (NMDARs). During ischemic stroke, reduced brain concentrations of oxygen and glucose can trigger increased neuronal calcium influx, a process that results in enhanced release of glutamate, the prototypical excitatory neurotransmitter. Excessive synaptic accumulation of glutamate is associated with neuronal injury and/or death, a pathological condition known as excitotoxicity. Pharmacological inhibition of NMDARs is known to protect against excitotoxicity-induced neuronal injury, which is the basis for the development of memantine as a neurotherapeutic. Memantine is a small molecule that can cross biological membranes by passive transcellular diffusion; however, it also carries a positive charge at physiological pH [138]. As such, memantine requires a selective membrane transport mechanism to be taken up into target tissues. Indeed, memantine has been shown to be a substrate for proton-coupled SLC transporters such as Oct1 and Oct2 [138]. Studies in *Xenopus laevis* oocytes transfected with OCT2 mRNA showed saturable uptake transport (K_m_ = 34 ± 5 μM) for memantine [139].

Metformin, an established OCT1 transport substrate [99], has also been shown to have neuroprotective properties. Clinically, metformin is widely known for its efficacy as a therapeutic for type 2 diabetes mellitus because of its ability to inhibit hepatic and peripheral glucose formation [140,141]. More recently, metformin has gained considerable interest as a potential therapeutic for ischemic stroke [142]. Indeed, metformin can permeate the BBB following a single oral dose of 50–150 mg/kg [142,143]. Beneficial effects in the setting of ischemic stroke for metformin are believed to result from activation of AMPK signaling, which can attenuate neuroinflammation, reduce oxidative stress, decrease apoptosis, promote physiological mitochondrial function, reduce glutamatergic excitotoxicity, and stimulate autophagy [142]. While the exact mechanisms for metformin and memantine transport across the BBB require more rigorous evaluation, the therapeutic targeting of neuroprotective drugs to the CNS via OCT-mediated drug delivery may, however, prove to be an effective mechanism to optimize pharmacotherapy of ischemic stroke.

## 3. Transporter Studies for Ischemic Stroke—How to Advance the Field?

Evaluation of endogenous BBB transporters in the setting of ischemic stroke requires careful selection of both a preclinical animal species and an appropriate in vivo stroke model. While animal studies have enabled the identification of novel molecules with properties that could potentially improve stroke pharmacotherapy, there has been minimal translation to successful human clinical trials. Similarly, in vivo models of ischemic stroke have clear advantages and disadvantages with respect to the study of drugs, which are often not properly considered during experimental design. Indeed, the lack of translation from animals to humans stems from a failure to properly consider the dynamics of individual preclinical species and/or the utility of the stroke model employed for the evaluation of drug pharmacokinetics and/or pharmacodynamics. Clearly, the greatest potential for translation of preclinical stroke studies comes from careful consideration of species and ischemic stroke models so that they both have relevance and resemblance to human stroke. The inclusion of state-of-the-art knowledge on transporter pharmacology in preclinical stroke studies will serve to further accelerate their translational potential. In this section, we highlight factors that should be considered when selecting an animal species or experimental stroke model for the study of BBB transporters.

### 3.1. Species Selection

#### 3.1.1. Application of Non-Human Primates to Preclinical Stroke Research

The Stroke Therapy Academic Industry Roundtable (STAIR) suggests the utilization of at least two in vivo model systems when exploring the efficacy of new and/or repurposed therapeutics for ischemic stroke: ideally, these studies should be conducted first in rodents and then in a larger animal model [144]. Non-human primates are advantageous for stroke research because their white matter content and cerebral volume are comparable to human brain tissue, although subtle differences in cerebral vascular anatomy and physiology exist. The most common gyrencephalic (i.e., possessing a cerebral cortex with convolutions) non-human primates used for modeling ischemic stroke are the *Papio* and *Macaca* species of marmoset [145]. Common approaches for inducing an infarct in non-human primates include craniotomy with electrocoagulation, pterional craniotomy, transorbital approaches, or embolization [146]. A recently developed technique involving endovascular transient occlusion of the middle cerebral artery (MCA) with PET-MRI and neuro-functional assessment has been reported to successfully induce focal cerebral ischemia in *Macaca fascicularis* [147]. Although the cerebral vasculature of marmosets and humans are comparable, it has been observed that the *Macaca* species is missing an anterior communicating artery [148]. *Macaca mullata* has the presence of a single distal anterior cerebral artery, thereby in direct contrast with human anatomy [149]. Other limitations of using non-human primates to model cerebral ischemia include the high cost of husbandry and the need for specialized surgical equipment and post-operative recovery requirements [146].

The application of non-human primates to the study of drug delivery in stroke presents unique challenges. Specifically, there is a dearth of published information on transporter localization, expression, and transport activity at the BBB in these species. Terasaki and colleagues attempted to address this critical knowledge gap by examining transporter abundance in brain capillaries isolated from marmosets using quantitative targeted proteomics [150]. Data from this study reported measurable protein abundance of a select panel of ABC transporters (i.e., P-gp, BCRP, MRP4) and SLC transporters (i.e., excitatory amino acid transporter 1 (EAAT1), glucose transporter (GLUT) 1 and 3, 4F2 heavy chain (SLC3A2), and monocarboxylate transporters 1 (MCT1)) at the marmoset BBB [150]. Furthermore, expression levels of the specific transporters that were detected showed a reasonable correlation with transporter expression data obtained from human brain capillaries [150]. It is important to point out that Hoshi and colleagues failed to detect several transporters in marmoset capillaries that are well-known to be expressed at the human BBB. Perhaps the most striking example is OATP1A2, which has been shown to be highly expressed in human brain capillaries isolated from frontal cortical brain tissue [122]. Functional studies on transporters at the BBB in non-human primates have primarily been restricted to the study of ABC transporters. For example, PET studies in baboons demonstrated that P-gp is a critical determinant of CNS penetration of [^11^C]metoclopramide [151]. More recently, P-gp inhibition with tariquidar (8 mg/kg) resulted in increased brain distribution of [^18^F]MC225, a probe substrate designed for evaluation of P-gp transport function in non-human primates [152]. Clearly, functional data on SLC transporters in non-human primates are required to advance the utilization of this species for studies on CNS drug delivery of neuroprotective drugs for ischemic stroke.

#### 3.1.2. Application of Rodents (i.e., Mice and Rats) to Preclinical Stroke Research

Rodents are the preferred animal model for preclinical research in ischemic stroke. While it is true that data from rodent studies have often lacked translatability from preclinical experiments to human patients in clinical trials, these concerns can be alleviated by careful experimental design and the use of a stroke model that correlates with the desired study endpoints. Rodents offer many advantages in preclinical stroke research: they require less room for equipment, have lower associated costs, and are easier to use for the development of ischemic stroke models than larger animal species. It is for these reasons that the majority of preclinical studies utilize rodents instead of non-human primates. The primary limitation of rodent in vivo models is that they have vast differences relative to human physiology and anatomy. Rodent brains are primarily lissencephalic (i.e., smooth cerebral cortices that lack convolutions) and are comprised of only 10% white matter. This differs considerably from human brains that are composed of more than 50-60% white matter [153,154,155]. In humans, subcortical white matter strokes account for almost 25% of ischemic stroke subtypes. The lack of white matter in rodent brain tissue renders these strokes difficult to replicate in these in vivo model systems. Anatomical variations in the cerebral vasculature exist between humans, rats, and mice. Specifically, the Circle of Willis in humans and rats distributes blood flow collaterally but does not do so in some strains of mice (i.e., ddY) [156]. Instead, blood flow is directed towards the olfactory artery and away from the internal carotid artery and, by extension, the middle cerebral artery [156]. Additionally, only 1 in 10 B6 mice have an intact Circle of Willis, 60% are absent a single posterior communicating artery, and 30% are missing both posterior communicating arteries [157]. These anatomical differences amongst rodents highlight a surprising variability in cerebral vascular anatomy, a factor that can greatly affect the utility of specific strains of rodents in ischemic stroke research. Furthermore, vascular anatomical differences can greatly affect preclinical data on CNS drug delivery, particularly when recanalization is incorporated into the study design.

Knowledge on transporter localization and functional expression is far more extensive in rodents as compared to any other preclinical species that can be used in ischemic stroke research. Rodent models are known to express all clinically relevant efflux transporters including P-gp, Bcrp, and various Mrp isoforms [20,24,69]. Interestingly, Uchida and colleagues reported that transporter protein expression tends to be higher at the rodent BBB as compared to the human BBB [158]. For example, P-gp was approximately 25-fold higher in capillaries isolated from rat whole cerebrum as compared to capillaries derived from human brain white matter [158]. Similarly, Bcrp at the rodent BBB was shown to be approximately 7-fold higher than BCRP protein levels at the human BBB [158]. These data imply that rodent models may exhibit higher rates of transport in cerebral microvasculature as compared to human tissues. Another critical consideration is species differences in transporter specificity. As noted previously in this review, it is critical to know whether a drug is a transport substrate for human and/or rodent transporter isoforms prior to initiating experimental work in rodent models. Specifically, if a drug is a substrate for a rodent transporter but not its human correlate (or vice versa), then BBB transport data will not translate into human studies. A good example of this principle is highlighted by our laboratory’s experience with statins. Our work has shown that currently marketed statins, drugs with known neuroprotective properties in ischemic stroke, are transport substrates for both Oatp1a4 [119,120,122] and its human orthologue OATP1A2 [100]. Such data emphasizes the translational potential of our transporter research program and has informed our advancement of statins into preclinical studies using in vivo models of focal cerebral ischemia.

### 3.2. Model Selection

#### 3.2.1. Focal Cerebral Ischemia (i.e., Middle Cerebral Artery Occlusion (MCAO))

One of the most established methods of inducing a cerebral infarction in an experimental animal is the middle cerebral artery occlusion (MCAO) model of focal cerebral ischemia [159,160,161]. This model requires the insertion of an intraluminal filament (often silicone-coated) into the animal’s vasculature to occlude blood flow from the middle cerebral artery (MCA), a common occlusion site associated with clinical cases of ischemic stroke. The surgery requires isolation of the common, external, and internal carotid arteries (CCA, ECA, ICA, respectively). The occipital and sternal arteries are cauterized, and thread is used to ligate the distal and proximal ends of the ECA around a small incision by the bifurcation. A intraluminal filament is then inserted and guided into the ICA to occlude the MCA. There are many variations of the MCAO model that are reported throughout the scientific literature. Common differences may include occlusion time, which typically ranges from 30–120 min transient (tMCAO) or permanent (pMCAO) occlusions [162]. The MCAO model yields reproducible infarct volumes that possess both an ischemic core and penumbra [146]. As such, this is a suitable model for neuroprotection research and is often utilized in the study of BBB damage and repair. Additionally, the MCAO model has been used to study pathological transport processes at the BBB in stroke such as perturbations of ion transporters [26,27,163,164,165] or glucose transporters [25,166,167]. While MCAO has not yet been extensively used to evaluate CNS delivery of neuroprotective drugs to the ischemic brain via putative BBB transporters for small molecules, this model has the utility to enable the acquisition of such valuable information.

Although a valid and reliable model of focal cerebral ischemia, the MCAO model does have known limitations. Specifically, this method can cause a greater degree of cerebral injury as compared to what is normally observed in ischemic stroke. For example, there have been reports in the MCAO model in which cerebral infarctions have extended into the hippocampus, substantia nigra, and thalamus [146]. Likewise, it has been shown that 60 min filamentous MCAO (fMCAO) with reperfusion can lead to retinal cell death; however, visual disturbances have been reported with stroke in humans so this retinal injury from tMCAO may be beneficial for those interested in studying retinal damage in patients [168]. Finally, reperfusion in the MCAO model does not accurately mimic restoration of cerebral blood flow following clinical ischemic stroke. Firstly, removal of the intraluminal filament allows for immediate reperfusion of the MCA, which is unlike the slower reperfusion due to thrombolysis. This event yields a response that resembles a global ischemic phenotype, which differs substantially from human stroke [161,169]. Specifically, vessel occlusion in human stroke is often not complete and spontaneous reperfusion can occur in many patients within the first 48 h post-stroke, an event that does not occur in the MCAO model with ad hoc reperfusion [169]. Nonetheless, MCAO has utility in the evaluation of BBB mechanisms in the setting of an ischemic insult as well as pathophysiological processes (i.e., inflammatory, cell death mechanisms) associated with stroke [169] Secondly, the MCAO is not an ideal model for evaluating fibrinolytic treatments since the occlusion is induced by a filament and not by a thrombus. To study the preclinical efficacy of these treatments for ischemic stroke, it is recommended to utilize a thrombotic cerebrovascular occlusion model.

#### 3.2.2. Embolic Stroke

Embolic stroke models are a more general category of preclinical models and do not require craniotomy. These are similar to thrombotic occlusion but utilize endogenous agents such as nylon thread, sutures, cyanoacrylate adhesive, polystyrene spheres, balloons, or microcatheters to impair cerebral arterial blood flow [170]. If it is desired to generate a stroke model that best mimics the pathophysiology of a naturally occurring cerebrovascular occlusion (i.e., a thrombus), it is preferable to utilize a thrombotic stroke model. Thromboembolic stroke model methodologies vary but typically include utilizing a catheter to administer autologous blood clots or clotting agents to occlude the middle cerebral artery [171,172,173,174,175,176]. This model allows for the use of recombinant tissue plasminogen activator (rt-PA) to induce reperfusion and, therefore, can enable the evaluation of neuroprotective agents to be given in conjunction with fibrinolytics. Many thrombotic stroke studies alter the size and number of clots to produce a better representative stroke model; however, depending on these factors, the outcome may result in small and variable cerebral infarct volumes making it difficult to analyze beneficial effects from the administration of neuroprotective drugs [177]. Data from thromboembolic events and rt-PA treatment in this model indicate that the BBB undergoes ischemic changes that are comparable to those observed in the MCAO model [178]. This suggests that thromboembolic stroke models can be successfully utilized to study cerebrovascular injury and/or transport mechanisms for novel stroke therapeutics with neuroprotective properties.

#### 3.2.3. Photothrombotic Stroke

Another model that induces clot formation to occlude vessels is the photothrombotic method. Rose Bengal is a photosensitive dye that is injected into the peritoneal cavity and circulates throughout the systemic circulation. The animal’s skull is then irradiated by a laser with a wavelength of 532 nanometers. This causes activation of Rose Bengal dye and subsequent production of reactive oxygen species (ROS) as well as injury to cerebrovascular endothelial cells. The contact activation pathway of the coagulation cascade is induced and leads to platelet occlusion of the vessel in the region of laser irradiation [161,179]. Ischemic cell death then rapidly follows vascular injury. Alterations in the photothrombotic stroke model have been documented, including utilizing freely moving animals. This allows for studying parameters of modeling stroke in real time that may be altered by anesthesia [180,181].

An advantage of photothrombotic stroke models is the ability to directly manipulate the location where occlusion occurs. This results in a cerebral injury that is highly reproducible from animal to animal as well as a low rate of mortality [169]; however, it is often debated if this model is truly relevant for preclinical stroke research. In fact, many researchers argue that the photothrombotic approach may not be directly inducing platelet activation and, therefore, may be better suited strictly for the study of BBB injury mechanisms [182,183]. Additionally, the photothrombotic stroke method does not result in an ischemic penumbra, thereby rendering it inapplicable for studies on the effectiveness of neuroprotective agents [169]. This limitation has been addressed through the establishment of a photothrombotic “ring” stroke model that utilizes a ring illumination filter to allow for a salvageable region of tissue [184,185,186] but it remains unclear if this “ring” accurately represents a clinical penumbra [177].

#### 3.2.4. Endothelin-1 Induced Stroke

Similar to the photothrombotic stroke model, endothelin-1 (ET-1) allows for the induction of stroke in an awake animal. Although this model is often performed on rodents, it can also be used for experimental stroke in non-human primates [187]. The ET-1 stroke model involves an injection of ET-1 to the perivascular surface of the MCA. ET-1 is naturally generated by endothelial cells and binds to endothelin-A and endothelin-B receptors to function as a vasoconstrictor [188]. When exogenously supplemented in high concentrations at the MCA, severe vasoconstriction leads to occluded blood flow and infarcted brain tissue similar to that observed in the setting of human stroke. Abeysinghe and Roulston report in a detailed protocol [189] that the surgery begins by anesthetizing the subject and mounting it onto a stereotaxic frame. The scalp is then exposed, allowing for positioning of screws and an injector guide cannula (some protocols, comparable to that reported by Ansari and colleagues [190], additionally mount a laser Doppler probe to measure the drop in blood flow during the ET-1 injection). In rats, the guide cannula should be positioned directly over the subject’s right MCA with drill coordinates in rats of anterior/posterior +0.9, medial/lateral −5.2, and dorsal/ventral −8.7. All components are then held in place with dental cement and the wound is sutured. Animals are given 4–5 days to recover prior to stroke induction in order to avoid influencing the assessment of functional neurological outcomes following stroke. To induce the stroke in a freely moving animal, a microinjector is loaded with ET-1 solution, inserted into the guide cannula, and steadily released at the site of the MCA. Some behavioral measures to assess stroke outcome in the ET-1-induced stroke model include the neurological deficit score, sensorimotor hemineglect, rotarod motor performance, cylinder test, and staircase test [189].

This method permits a less invasive surgery than other given stroke models (i.e., the MCAO). It is also relatively quick and, most importantly, harnesses certain aspects of human stroke. These include the level of consciousness in which a stroke occurs, as well as the spontaneity and gradual rate of reperfusion. Slower reperfusion more accurately represents the phenotype observed from natural thrombolysis [191]. Although this technique yields infarction volumes that are highly variable [190], some argue that this factor actually makes it a better system to model stroke due to the high variation of infarction volumes measured in human stroke patients [189]. Studies have demonstrated that the ischemic infarction generated from ET-1 also mimics stroke evolution in humans and, therefore, would be a reliable model for research on stroke therapeutics [192]; however, the lack of transporter studies in the ET-1 model makes it difficult to rely on it for drug research. In fact, there is limited research on how the specifics of ET-1 as a stroke model may alter transporter expression and localization at the BBB. Although, it has been reported that endogenous release of ET-1 in the brain (i.e., not localized to the MCA) causes increased P-gp transport activity [193,194]. Harati and colleagues measured P-gp and BCRP transport after intracerebroventricular injections of ET-1 and found enhanced activity of both P-gp and BCRP in adult rats [195]. Knowledge of altered drug transport due to supplemented ET-1 at the site of the MCA is critical for predicting translatability of drug permeability in humans. If more information can be obtained regarding transporter alterations in ischemic stroke models, optimized approaches can be taken to utilize a model that best represents both human stroke phenotypes and functional transporter expression.

## 4. Role of Co-Morbidities in Preclinical Transporter Studies in Stroke

The expression of both ABC and SLC transporters at the BBB implies a highly complex system that regulates the permeation of drugs into the CNS. Many pathologies that are associated with altered expression of drug transport proteins are also known to increase the risk of ischemic stroke and/or to worsen functional neurological outcomes during the acute, subacute, and chronic phases of post-stroke recovery. Additionally, each of the following conditions may alter transporter levels and mechanisms in a way that could change drug delivery to ischemic brain tissue. The design of preclinical studies to evaluate BBB transport processes in the setting of ischemic stroke requires an appreciation of how transporter expression and/or activity may be altered by these comorbid conditions. Such considerations are critical to the interpretation of transporter data obtained from preclinical stroke models and will enable the results of these experiments to optimize their relevance to human stroke.

### 4.1. Diabetes Mellitus

Diabetes mellitus (DM) is a well-established risk factor for both ischemic and hemorrhagic stroke. In the United States, 65% of deaths from DM were ascribable to either stroke or cardiovascular disease (CVD) [196]. Interestingly, individuals with type II DM are more likely to experience stroke than those with type I DM [197]. It has also been noted that DM increases prevalence rates of ischemic stroke at all ages, although this co-morbidity is most pronounced in the younger age groups (i.e., those aged 55 and under and 65 and under for Black and White populations, respectively) [198]. It is also noteworthy that a greater risk of ischemic stroke is linked to pre-DM or to impaired glucose tolerance [199]. The exact mechanism by which DM increases stroke risk is unknown but may include injury to vascular endothelial cells, thickening of the basement membrane in cerebral capillaries, or enhanced arterial stiffness at a younger age [200]. Studies in the BBZDR/Wor rat, an animal model of type II DM, have shown an increase in blood-to-brain permeability to ferumoxytol in over 84% of cerebral microvessels as well as global capillary pathology [201]. Using the streptozotocin model, Hawkins and colleagues showed that BBB dysfunction in DM is associated with increased matrix metalloproteinase (MMP) activity and decreased expression of critical tight junction proteins such as occludin and ZO-1 [202]. Activation of MMPs can be triggered by vascular endothelial growth factor (VEGF), a signaling protein whose release is stimulated by advanced glycation end products [203]. Systemic inflammation in DM is also a critical causative factor that promotes BBB injury and risk of ischemic stroke [196]. Additionally, oxidative stress secondary to glucose processing via the Kreb’s cycle can lead to vascular pericyte loss and, subsequently, increased BBB “leak” [203]. It is also critical to note that obesity is often comorbid with DM and can also promote BBB dysfunction. Using a murine obese model of diet-induced type II DM, Salameh and colleagues showed that high-fat feeding increased BBB permeability to [^14^C]sucrose and [^99m^Tc]albumin [204]. At the molecular level, reduced microvascular expression of claudin-5, claudin-12, occludin, and ZO-1 was observed in animals fed a high-fat diet as compared to low-fat feeding controls [204]. Further advancement in this field is critical for understanding novel targets and developing effective pharmacological treatments and preventatives for diabetes-induced stroke [205,206].

Several studies are paving the way to improve our understanding of the cellular and molecular effects of DM on the BBB. Included amongst these studies is the examination of membrane transport mechanisms in brain microvasculature. Of particular note, studies focusing on alterations in glucose transport may provide new information on mechanisms associated with BBB disruption. The brain requires the highest consumption of glucose-derived energy relative to other organs, approximately 20% of total glucose, even though it only represents approximately 2% of total body mass [207]. Therefore, having sufficient glucose transport at the BBB is critical to allow cells such as neurons and astrocytes to properly function. Glucose can traverse the BBB into the brain by means of facilitative glucose transporters, the most highly expressed isoform being GLUT-1/Glut-1 (*SLC2A1*). This uniporter is localized to both the luminal and abluminal membranes of the microvascular endothelium and functions by moving glucose from the blood into brain tissue along its concentration gradient. Differences in BBB endothelial cell expression and localization of GLUT-1/Glut-1 across animals have previously been reported in the scientific literature [208,209,210,211,212,213,214]. More recently, proteomic analysis confirmed higher rates of binding and transport at the plasma luminal membrane of porcine brain capillaries [75]. Some studies have shown decreased glutamate transporter protein expression during DM; however, other data have not supported this finding [205], thereby highlighting inconsistencies on how DM may play a role in altering BBB transporters. In the setting of focal cerebral ischemic, numerous studies have shown upregulation of Glut-1 in in vivo rodent models [215,216,217,218]. Of particular significance, Zhang and colleagues found that these results are more pronounced in streptozotocin-induced diabetic rats subjected to 90 min MCAO with perfusion times of 3, 12, 24, and 72 h [219]. With respect to ABC transporters, in vitro and in vivo studies have provided limited and conflicting evidence for altered expression and/or transport activity in the setting of DM. Studies in primary cultures of rat brain endothelial cells showed that P-gp functional expression is reduced following 72 h exposure to serum isolated from diabetic rats [220]. In contrast, P-gp-mediated transport of cyclosporine A, an established substrate of this ABC transporter, was increased at the BBB in the streptozotocin-induced DM rat model [221]. P-gp functional expression was also reported to be increased in striatal capillaries isolated from obese and type II DM New Zealand mice [222]. In summary, these observations provide evidence for altered transporter function at the BBB in DM, a factor that must be considered when incorporating this disease as a comorbidity in preclinical stroke studies.

### 4.2. Tobacco Smoking

Approximately 20% of the US adult population smokes tobacco products [223], according to the Center for Disease Control and Prevention (CDC). Numerous studies have demonstrated a strong relationship between smoking and the development of ischemic stroke [224,225,226,227]. In fact, tobacco smoking is one of the most prominent risk factors for ischemic stroke. Pan and colleagues published a meta-analysis on 14 independent studies and found a significant increase in stroke risk amongst smokers as compared to nonsmokers. Interestingly, they also noted that every increment of five cigarettes per day resulted in an increased risk of stroke by 12% [228]. Similarly, e-cigarettes, such as vaping products, have been linked to an amplified risk of stroke with their flavorings also playing a potential role in endothelial cell dysfunction [229]. Furthermore, products that emit secondhand smoke (SHS) including cigarettes, cigars, and e-cigarettes may have a profound impact on the risk of stroke in a non-smoker. Malek and colleagues reported that the incidence of stroke in adult non-smokers who were exposed to SHS increased by 30% after adjustment of other stroke risk factors [230]. There is even an increased risk of stroke over the age of 45 in non-smokers if SHS was present in childhood [231]. SHS has been linked to an increase in carotid artery intimal-medial wall thickness, which is indicative of atherosclerosis [232].

Possible mechanisms in which both SHS and primary exposure can increase stroke risk include atherosclerosis, reduced plasma concentrations of HDL-cholesterol, carboxyhemoglobinemia, increased platelet aggregability, and increased fibrinogen levels [233]. Development of some of these factors (i.e., atherosclerosis) can be directly linked to compounds in tobacco, such as 1,3-butadiene. This vapor in tobacco smoke has been linked to arteriosclerosis in animal models at an environmentally relevant dose [234]. The release of inflammatory mediators is another mechanism that may contribute to stroke risk and be induced from smoking [228]. The release of ROS from smoking that causes inflammation also promotes considerable BBB impairment [235]. Nicotine from tobacco smoke leads to increased BBB permeability through disruption of endothelial cell tight junctions; specifically, this is thought to occur due to a modified distribution of tight junction scaffolding protein zonula occludens-1 (ZO-1) [236,237,238]. The integrity of the cerebral microvasculature can be further compromised if a smoking-induced increase in blood viscosity occurs in tandem with BBB tight junction disruption [239]. Similarly, nicotine binds to nicotinic acetylcholine receptors causing nitric oxide (NO) release and a cascade of physiologic events that ultimately enhance BBB permeability. Studies have also indicated that transporters at the BBB can be affected by nicotine found in tobacco smoking, including ion transporters and P-gp [240]. Studies from the Abbruscato laboratory have shown that nicotine in tobacco products hinders the activity of Na-K-2Cl co-transporters on the abluminal surface of the BBB, resulting in decreased K+ uptake in stroke conditions [241]. Meanwhile, data from Manda and colleagues have indicated that nicotine is linked to inhibition of P-gp transport at the BBB [242].

### 4.3. Atrial Fibrillation

Many factors can contribute to the development of uncoordinated heart rhythms, often diagnosed as atrial fibrillation (AF), including age, hypertension, heart disease, and genetic components. Uncoordinated rhythms from the heart’s upper and lower chambers can result in arrhythmia, poor blood flow, and even fibrosis with blood clot formation. When the latter occurs in AF, there is an elevated risk of ischemic stroke. In fact, there is such a high risk of stroke with AF that roughly 30% of all strokes take place in AF patients [243]. Unfortunately, those who have this co-morbidity tend to have higher rates of disability and mortality from experiencing a stroke as compared to those who do not have AF [244]. Additionally, individuals with AF have a 21.5% chance of experiencing a secondary stroke within five years of their initial stroke [245]. Although anticoagulant drugs, such as warfarin, limit the risk of stroke [246], many individuals are not aware that they have AF and, therefore, have not been prescribed these preventative measures.

Reports have indicated that AF can disrupt the BBB, specifically through abnormal cerebral blood flow. Disrupted cerebral autoregulation and neurovascular coupling that leads to this irregular blood flow [247] are often linked to the BBB disruption found in ischemic stroke [248]. Many studies have detected a reduction in brain blood flow in patients and animal models of AF [249,250,251]. Aryal and Patabendige recently published a review that goes into detail explaining the mechanisms and methods of cerebral blood flow in AF and its relevance to stroke [252]. Although it appears likely that AF may disrupt the BBB, its mechanism of action needs further assessment. Likewise, investigating how AF may affect transporters at the BBB is crucial to gaining a better understanding of BBB integrity and possible neuroprotective treatments to reduce stroke likelihood. At present, it is unknown if AF can modulate transport processes at the brain microvascular endothelium.

### 4.4. Hypertension

High blood pressure is considered to be the most robust modifiable risk factor for ischemic stroke. This is concerning due to the vast population of Americans who have hypertension, which is estimated to be approximately 30% [253]. In a study by Ostwald, Wasserman, and Davis, 86.5% of their study population of 97 stroke survivors also had a co-morbidity of hypertension [254]. Those who experience hypertension during pregnancy (i.e., preeclampsia) are 60% more likely to have a stroke later in life [255]. Not only is hypertension a risk factor for stroke occurrence, but it also increases the severity of the stroke outcome. Hypertension can lead to a larger infarct with a smaller penumbra, implying a significant decrease in the quantity of brain tissue that can be salvaged by neuroprotective measures [256]. Consistently elevated blood pressure causes vascular injury by means of increased mechanical and shear stress. Increased blood flow can lead to elastin fiber degradation and artery stiffening through mechanical stress and increased force on and resulting dysfunction of endothelium through shear stress. Typically, shear stress is adaptive by increasing NO production; however, since hypertension damages endothelial cells, the ability to produce physiological NO diminishes. This can lead to an upregulation of atherogenic genes and, ultimately, atherosclerosis which plays a large risk factor for vessel occlusion [256,257].

A study using an acute hypertensive rat model found that tight junction claudin mRNA in endothelial cells was reduced, leading to a disruption of the BBB [258]. A recent study by Nakagawa et al. also reported decreased claudin-5 expression in brain endothelial cells from a spontaneous hypertensive rat model prone to stroke (SHRSP) that has a functionally defective BBB. They co-cultured brain-derived endothelial cells, pericytes, and astrocytes from SHRSP rats and compared them to co-cultures from control Wistar Kyoto rats. Their data suggest that BBB dysfunction in the hypertensive model could be contributed to a defective interaction between brain endothelial cells and astrocytes. Finally, the authors measured the expression of BBB transporters by Western blot and revealed that SHRSP endothelial cells had decreased Glut1 and increased P-gp protein levels, suggesting altered BBB functionality in the hypertensive model [259]. Additionally, BBB expression of NHE1 was shown to be enhanced at the BBB in 15-week-old spontaneously hypertensive rats, suggesting that a pervasive increase in systemic blood pressure can cause profound cerebral vascular changes including altered functional expression of transport proteins [260].

### 4.5. Aging

While hypertension is the most robust modifiable risk factor for stroke, aging is the most non-modifiable. The risk of ischemic stroke incidence doubles every ten years after age 55. In fact, individuals over age 65 represent nearly 75% of all strokes [261]. Older stroke patients are likely to have other co-morbidities as well; there is an estimated 89% of people 65 or older with an incidence of stroke who have multi-morbidities [262]. Suenaga and colleagues have indicated that stroke in aged subjects leads to greater infarct volumes and enhanced neurological dysfunction [263]. Stroke risk in the aged population may be attributed to the natural aging processes. Senescence can impair angiogenesis, decrease circulation in the microvasculature, and initiate atherosclerosis.

Similarly, age-related dysfunction of the BBB begins in adulthood and continues due to inflammation and loss of tight junctions [264]. In endothelial cells, age-induced mitochondrial dysfunction can affect cerebral blood flow regulation and transporter function. Cerebral blood flow becomes dysregulated by impaired vasodilation due to the decline of vasodilators (i.e., NO) [265]. Additionally, reduced function of NADPH-oxidase 2 (NOX2) following pharmacological inhibition with apocynin increased mortality rate and worsened BBB dysfunction in aged rats subjected to 120 min embolic stroke via autologous blood clot with thrombolysis induced by r-tPA treatment [266]. Although senile brain endothelial cells play a large role in the disruption of vasculature growth and repair, many other mechanisms, such as decreased secretion of growth factors and circulating endothelial progenitor cells with age, contribute to the arrest of angiogenesis [267,268]. Impaired mitochondria in endothelial cells may also contribute to the dysfunction of the aged BBB by disrupting the normal functioning of ATP-dependent active transporters (i.e., ABC and Na^+^ K^+^ ATPase) [269]. Wu and colleagues demonstrated that P-gp expression elevates with age using senescence-accelerated mice with age-related deficits [270]. Understanding the effects that age has not only on transporter mechanisms but also on functional neurocognitive outcomes is essential for improving pharmacological treatments and/or developing novel therapeutic paradigms for stroke.

## 5. Summary and Conclusions

Ischemic stroke continues to be one of the most significant causes of morbidity and mortality in the United States. At present, FDA-approved drug treatments for ischemic stroke are limited to fibrinolytic therapy with rt-PA. Although recanalization of infarcted brain tissue is critical, adverse events associated with r-tPA treatment (i.e., bleeding complications) are serious and can even exacerbate neurological/vascular injury and worsen post-stroke neurological deficits. This indicates a need for stroke therapeutics that are both safe and effective as neuroprotectants. The clinical utility of neuroprotective compounds is highly dependent upon efficient transport from the blood into ischemic brain tissue. Previous research has revealed several endogenous transporters that can be targeted to optimize CNS delivery of neuroprotective therapeutics; however, there are few studies on transport mechanisms for such drugs in the preclinical stroke literature. In fact, the vast majority of transport studies in the setting of stroke have been limited to those membrane transport mechanisms that contribute to pathophysiological injury such as ion transporters and glucose transporters. Indeed, preclinical stroke research will take a “giant step forward” via the incorporation of drug transporter experimentation into in vivo models of ischemic stroke in an effort to understand how drugs can attain therapeutic concentrations in ischemic brain tissue and to identify what transporters allow them to achieve these efficacious concentrations. It is noteworthy that statins and memantine, drugs that have shown varying degrees of clinical success in stroke, are substrates for uptake transporters that are expressed at the human brain microvascular endothelium (i.e., OATP1A2 for statins; OCT1/OCT2 for memantine). Future development of neuroprotective treatment strategies for ischemic stroke will greatly depend upon obtaining a rigorous understanding of BBB transport mechanisms. Information derived from BBB transport studies can then be extended to inform the discovery of new chemical entities developed specifically for the treatment of ischemic stroke. Success in this area will also depend upon the selection of an appropriate preclinical stroke model and the consideration of co-morbid conditions (i.e., DM, smoking, atrial fibrillation, hypertension, aging, etc.) that affect functional neurological outcomes in the acute, subacute, and chronic phases post-stroke. Overall, endogenous transporters at the BBB represent an untapped opportunity to accelerate the development of pharmacological strategies for the treatment of ischemic stroke.

## Figures and Tables

**Table 1:** Confirmed localization of ATP-Binding Cassette (ABC) and Solute Carrier (SLC) transporters in brain microvascular endothelial cells across preclinical animal species.

Transporter	Mouse	Rat	Porcine	Bovine	Non-Human Primate	Human
**OATPs/Oatps (SLCOs/Slcos)**	Oatp1a4: Luminal and Abluminal [71]	Oatp1a4: Luminal [72]; Luminal and Abluminal [73] Oatp1c1: Luminal and Abluminal [74] Oatp2b1: Abluminal [72]	Oatp3a1: Primarily Luminal [75]	Not defined	Not defined	OATP1A2: Luminal and Abluminal [76,77,78] OATP2B1: Luminal [79]
				
				
**OCTs/Octs (SLC22A/Slc22a)**	Oct1 and Oct2: Primarily Luminal [80,81]	Oct1 and Oct2: Primarily Luminal [81]	Not defined	Not defined	Not defined	OCT1 and OCT2: Primarily Luminal [80,81]
**MATE1/Mate1 (SLC47A1/Slc47a1)**	Not defined	Not defined	Not defined	Not defined	Not defined	Not defined
**P-gp (MDR1/Mdr1)** **(ABCB1, Abcb1a/Abcb1b)**	Luminal [82]	Luminal [83]; Luminal and Abluminal [84]	Luminal [75]	Luminal [85]	Luminal [86]	Luminal [87]; Luminal and Abluminal [84]
**BCRP/Bcrp (ABCG2/Abcg2)**	Luminal [88]	Primarily Luminal [89]	Primarily Luminal [75]	Not defined	Not defined	Primarily Luminal [90]
**MRP1/Mrp1 (ABCC1/Abcc1)** **MRP4/Mrp4 (ABCC4/Abcc4)** **MRP5/Mrp5 (ABCC5/Abcc5)**	Abluminal [82,91] Luminal [92] Luminal [82]	Abluminal [72] Luminal [72] Luminal [72]	Luminal [93] Not defined Luminal and Abluminal [75]	Luminal [94] Luminal and Abluminal [94] Luminal [94]	Not defined Not defined Not defined	Luminal [95] Luminal [79,95] Luminal [79,95]

**Table 2:** Representative transport substrates for ATP-Binding Cassette (ABC) and Solute Carrier (SLC) transporters at the Blood-Brain Barrier (BBB).

Transporter	Representative Centrally Active Transport Substrates
**OATPs/Oatps** **(SLCOs/Slcos)**	HMG CoA Reductase Inhibitors (i.e., statins; atorvastatin, pravastatin, rosuvastatin) [5,96] Prostaglandin E_2_ [96] Estrone-3-Sulfate, Dihydroepiandrosterone Sulfate (DHEAS), Estradiol-17β-Glucuronide [97]
**OCTs/Octs** **(SLC22A/Slc22a)**	Memantine [5] Pramipexole, Selegiline, Varenicline [6] Amisulpride [98] Metformin [99]
**MATE1/Mate1** **(SLC47A1/Slc47a1)**	Metformin, Thiamine, Topotecan [96] Amisulpride [98]
**P-gp (MDR1/Mdr1)** **(ABCB1/Abcb1a/Abcb1b)**	Amitriptyline, Cyclosporine A, Lapatinib, Losartan, Lovastatin, Phenytoin, Tetracycline, Verapamil [96] Atorvastatin, Rosuvastatin [100] Tacrolimus, Rifampicin [29]
**BCRP/Bcrp** **(ABCG2/Abcg2)**	Coumestrol, Daidzein, Dantrolene, Dipyridamole, Estradiol-17β-Glucuronide, Genistein, Glyburide, Lapatinib [96] Atorvastatin, Pravastatin, Rosuvastatin [100] Estrone-3-Sulfate, Dihydroepiandrosterone Sulfate (DHEAS), Dihydrotestosterone (DHT) [97]
**MRPs/Mrps** **(ABCC/Abcc)**	Glutathione (GSH), GSH Conjugates, GSSG [5] Estradiol-17β-Glucuronide [96] Estrone-3-Sulfate, Dihydroepiandrosterone Sulfate (DHEAS) [97]

## Data Availability

Not applicable.

## References

[1] Feigin V.L., Stark B.A., Johnson C.O., Roth G.A., Bisignano C., Abady G.G., Abbasifard M., Abbasi-Kangevari M., Abd-Allah F., Abedi V. (2021). Global, regional, and national burden of stroke and its risk factors, 1990–2019: A systematic analysis for the Global Burden of Disease Study. Lancet Neurol..

[2] Virani S.S., Alonso A., Aparicio H.J., Benjamin E.J., Bittencourt M.S., Callaway C.W., Carson A.P., Chamberlain A.M., Cheng S., Delling F.N. (2021). Heart Disease and Stroke Statistics-2021 Update: A Report From the American Heart Association. Circulation.

[3] Liu S., Levine S.R., Winn H.R. (2010). Targeting ischemic penumbra: Part I—From pathophysiology to therapeutic strategy. J. Exp. Stroke Transl. Med..

[4] Manning N.W., Campbell B.C.V., Oxley T.J., Chapot R. (2014). Acute ischemic stroke: Time, penumbra, and reperfusion. Stroke.

[5] Brzica H., Abdullahi W., Ibbotson K., Ronaldson P.T. (2017). Role of Transporters in Central Nervous System Drug Delivery and Blood-Brain Barrier Protection: Relevance to Treatment of Stroke. J. Cent. Nerv. Syst. Dis..

[6] Williams E.I., Betterton R.D., Davis T.P., Ronaldson P.T. (2020). Transporter-Mediated Delivery of Small Molecule Drugs to the Brain: A Critical Mechanism That Can Advance Therapeutic Development for Ischemic Stroke. Pharmaceutics.

[7] The National Institute of Neurological Disorders and Stroke (1995). rt-PA Stroke Study Group. Tissue plasminogen activator for acute ischemic stroke. N. Engl. J. Med..

[8] O’Carroll C.B., Rubin M.N., Chong B.W. (2015). What is the role for intra-arterial therapy in acute stroke intervention?. Neurohospitalist.

[9] Tymianski M. (2017). Combining neuroprotection with endovascular treatment of acute stroke: Is there hope?. Stroke.

[10] Shi L., Rocha M., Leak R.K., Zhao J., Bhatia T., Mu H., Wei Z., Yu F., Weiner S.L., Ma F. (2018). A new era for stroke therapy: Integrating neurovascular protection with optimal reperfusion. J. Cereb. Blood Flow Metab..

[11] Goyal M., Menon B.K., van Zwam W.H., Dippel D.W.J., Mitchell P.J., Demchuk A.M., Dávalos A., Majoie C.B.L.M., van der Lugt A., de Miquel M.A. (2016). Endovascular thrombectomy after large-vessel ischaemic stroke: A meta-analysis of individual patient data from five randomised Trials. Lancet.

[12] Nogueira R.G., Jadhav A.P., Haussen D.C., Bonafe A., Budzik R.F., Bhuva P., Yavagal D.R., Ribo M., Cognard C., Hanel R.A. (2018). Thrombectomy 6 to 24 Hours after Stroke with a Mismatch between Deficit and Infarct. N. Engl. J. Med..

[13] Pan J., Konstas A.-A., Bateman B., Ortolano G.A., Pile-Spellman J. (2007). Reperfusion injury following cerebral ischemia: Patho-physiology, MR imaging, and potential therapies. Neuroradiology.

[14] Candelario-Jalil E. (2009). Injury and repair mechanisms in ischemic stroke: Considerations for the development of novel neuro-therapeutics. Curr. Opin. Investig. Drugs.

[15] Eltzschig H.K., Eckle T. (2011). Ischemia and reperfusion—From mechanism to translation. Nat. Med..

[16] Nour M., Scalzo F., Liebeskind D.S. (2012). Ischemia-Reperfusion Injury in Stroke. Interv. Neurol..

[17] Abdullahi W., Tripathi D., Ronaldson P.T. (2018). Blood-brain barrier dysfunction in ischemic stroke: Targeting tight junctions and transporters for vascular protection. Am. J. Physiol.-Cell Physiol..

[18] Turner R.C., Lucke-Wold B., Lucke-Wold N., Elliott A.S., Logsdon A.F., Rosen C.L., Huber J.D. (2013). Neuroprotection for is-chemic stroke: Moving past shortcomings and identifying promising directions. Int. J. Mol. Sci..

[19] Kågedal M., Nilsson D., Huledal G., Reinholdsson I., Cheng Y.-F., Asenblad N., Pekar D., Borgå O. (2007). A Study of Organic Acid Transporter-Mediated Pharmacokinetic Interaction Between NXY-059 and Cefuroxime. J. Clin. Pharmacol..

[20] Sanchez-Covarrubias L., Slosky L., Thompson B., Davis T., Ronaldson P. (2014). Transporters at CNS Barrier Sites: Obstacles or Opportunities for Drug Delivery?. Curr. Pharm. Des..

[21] Hawkins B.T., Ocheltree S.M., Norwood K.M., Egleton R.D. (2007). Decreased blood-brain barrier permeability to fluorescein in streptozotocin-treated rats. Neurosci. Lett..

[22] Okamura T., Okada M., Kikuchi T., Wakizaka H., Zhang M.-R. (2020). Mechanisms of glutathione-conjugate efflux from the brain into blood: Involvement of multiple transporters in the course. J. Cereb. Blood Flow Metab..

[23] Li J., Zheng M., Shimoni O., Banks W.A., Bush A.I., Gamble J.R., Shi B. (2021). Development of Novel Therapeutics Targeting the Blood–Brain Barrier: From Barrier to Carrier. Adv. Sci..

[24] Abdullahi W., Davis T.P., Ronaldson P.T. (2017). Functional expression of P-glycoprotein and organic anion transporting polypep-tides at the blood-brain barrier: Understanding transport mechanisms for improved cns drug delivery?. AAPS J..

[25] Vemula S., Roder K.E., Yang T., Bhat G.J., Thekkumkara T.J., Abbruscato T.J. (2009). A Functional Role for Sodium-Dependent Glucose Transport across the Blood-Brain Barrier during Oxygen Glucose Deprivation. J. Pharmacol. Exp. Ther..

[26] Chen Y.-J., Yuen N., Wallace B.K., Wulff H., O’Donnell M.E. (2015). Blood brain barrier KCa3.1 channels: Evidence for a role in brain Na uptake and edema during ischemic stroke. Stroke.

[27] O’Donnell M.E. (2014). Blood–Brain Barrier Na Transporters in Ischemic Stroke. Adv. Pharmacol..

[28] Shah K.K., Boreddy P.R., Abbruscato T.J. (2015). Nicotine pre-exposure reduces stroke-induced glucose transporter-1 activity at the blood–brain barrier in mice. Fluids Barriers CNS.

[29] Spudich A., Kilic E., Xing H., Kilic Ü., Rentsch K.M., Wunderli-Allenspach H., Bassetti C.L., Hermann D.M. (2006). Inhibition of multidrug resistance transporter-1 facilitates neuroprotective therapies after focal cerebral ischemia. Nat. Neurosci..

[30] Cen J., Liu L., Li M.-S., He L., Wang L.-J., Liu Y.-Q., Liu M., Ji B.-S. (2013). Alteration in P-glycoprotein at the blood–brain barrier in the early period of MCAO in rats. J. Pharm. Pharmacol..

[31] Demars K.M., Yang C., Hawkins E.K., McCrea A.O., Siwarski D.M., Candelario-Jalil E. (2017). Spatiotemporal Changes in P-glycoprotein Levels in Brain and Peripheral Tissues Following Ischemic Stroke in Rats. J. Exp. Neurosci..

[32] Yemisci M., Caban-Toktas S., Gürsoy-Ozdemir Y., Lule S., Novoa-Carballal R., Riguera R., Fernandez-Megia E., Andrieux K., Couvreur P., Capan Y. (2015). Systemically Administered Brain-Targeted Nanoparticles Transport Peptides across the Blood—Brain Barrier and Provide Neuroprotection. J. Cereb. Blood Flow Metab..

[33] Bao Q., Hu P., Xu Y., Cheng T., Wei C., Pan L., Shi J. (2018). Simultaneous Blood–Brain Barrier Crossing and Protection for Stroke Treatment Based on Edaravone-Loaded Ceria Nanoparticles. ACS Nano.

[34] Lu X., Zhang Y., Wang L., Li G., Gao J., Wang Y. (2021). Development of L-carnosine functionalized iron oxide nanoparticles loaded with dexamethasone for simultaneous therapeutic potential of blood brain barrier crossing and ischemic stroke treatment. Drug Deliv..

[35] Montaner J., Cano-Sarabia M., Simats A., Hernández-Guillamon M., Rosell A., Maspoch D., Campos-Martorell M. (2016). Charge effect of a liposomal delivery system encapsulating simvastatin to treat experimental ischemic stroke in rats. Int. J. Nanomed..

[36] Al-Ahmady Z.S., Jasim D., Ahmad S.S., Wong R., Haley M., Coutts G., Schiessl I., Allan S.M., Kostarelos K. (2019). Selective Liposomal Transport through Blood Brain Barrier Disruption in Ischemic Stroke Reveals Two Distinct Therapeutic Opportunities. ACS Nano.

[37] Santos S.D., Xavier M., Leite D.M., Moreira D., Custódio B., Torrado M., Castro R., Leiro V., Rodrigues J., Tomás H. (2018). PAMAM dendrimers: Blood-brain barrier transport and neuronal uptake after focal brain ischemia. J. Control. Release.

[38] Zhang Y., Pardridge W.M. (2001). Neuroprotection in Transient Focal Brain Ischemia After Delayed Intravenous Administration of Brain-Derived Neurotrophic Factor Conjugated to a Blood-Brain Barrier Drug Targeting System. Stroke.

[39] Daneman R., Prat A. (2015). The blood-brain barrier. Cold Spring Harb. Perspect. Biol..

[40] Iadecola C. (2017). The Neurovascular Unit Coming of Age: A Journey through Neurovascular Coupling in Health and Disease. Neuron.

[41] DiNapoli V.A., Huber J.D., Houser K., Li X., Rosen C.L. (2008). Early disruptions of the blood–brain barrier may contribute to exacerbated neuronal damage and prolonged functional recovery following stroke in aged rats. Neurobiol. Aging.

[42] Sakadžić S., Lee J., Boas D.A., Ayata C. (2015). High-resolution in vivo optical imaging of stroke injury and repair. Brain Res..

[43] Winkler L., Blasig R., Breitkreuz-Korff O., Berndt P., Dithmer S., Helms H.C.C., Puchkov D., Devraj K., Kaya M., Qin Z. (2021). Tight junctions in the blood–brain barrier promote edema formation and infarct size in stroke–Ambivalent effects of sealing proteins. J. Cereb. Blood Flow Metab..

[44] Lochhead J., McCaffrey G., Quigley C.E., Finch J., Demarco K.M., Nametz N., Davis T.P. (2010). Oxidative Stress Increases Blood–Brain Barrier Permeability and Induces Alterations in Occludin during Hypoxia-Reoxygenation. J. Cereb. Blood Flow Metab..

[45] Hashimoto Y., Campbell M. (2020). Tight junction modulation at the blood-brain barrier: Current and future perspectives. Biochim. Biophys. Acta (BBA)—Biomembr..

[46] Abbott N.J., Patabendige A.A., Dolman D.E., Yusof S.R., Begley D.J. (2010). Structure and function of the blood-brain barrier. Neurobiol. Dis..

[47] Greene C., Hanley N., Campbell M. (2019). Claudin-5: Gatekeeper of neurological function. Fluids Barriers CNS.

[48] Lochhead J.J., Yang J., Ronaldson P.T., Davis T.P. (2020). Structure, Function, and Regulation of the Blood-Brain Barrier Tight Junction in Central Nervous System Disorders. Front. Physiol..

[49] Hashimoto Y., Campbell M., Tachibana K., Okada Y., Kondoh M. (2021). Claudin-5: A Pharmacological Target to Modify the Permeability of the Blood–Brain Barrier. Biol. Pharm. Bull..

[50] Nitta T., Hata M., Gotoh S., Seo Y., Sasaki H., Hashimoto N., Furuse M., Tsukita S. (2003). Size-selective loosening of the blood-brain barrier in claudin-5-deficient mice. J. Cell Biol..

[51] McCaffrey G., Seelbach M.J., Staatz W.D., Nametz N., Quigley C., Campos C.R., Brooks T.A., Davis T.P. (2008). Occludin oli-gomeric assembly at tight junctions of the blood-brain barrier is disrupted by peripheral inflammatory hyperalgesia. J. Neuro-chem..

[52] McCaffrey G., Willis C.L., Staatz W.D., Nametz N., Quigley C.A., Hom S., Lochhead J.J., Davis T.P. (2009). Occludin oligomeric assemblies at tight junctions of the blood-brain barrier are altered by hypoxia and reoxygenation stress. J. Neurochem..

[53] Pan J., Qu M., Li Y., Wang L., Zhang L., Wang Y., Tang Y., Tian H.-L., Zhang Z., Yang G.-Y. (2020). MicroRNA-126-3p/-5p Overexpression Attenuates Blood-Brain Barrier Disruption in a Mouse Model of Middle Cerebral Artery Occlusion. Stroke.

[54] Bhowmick S., D’Mello V., Caruso D., Wallerstein A., Abdul-Muneer P. (2019). Impairment of pericyte-endothelium crosstalk leads to blood-brain barrier dysfunction following traumatic brain injury. Exp. Neurol..

[55] Xia Y.-P., He Q.-W., Li Y.-N., Chen S.-C., Huang M., Wang Y., Gao Y., Huang Y., Wang M.-D., Mao L. (2013). Recombinant Human Sonic Hedgehog Protein Regulates the Expression of ZO-1 and Occludin by Activating Angiopoietin-1 in Stroke Damage. PLoS ONE.

[56] Seelbach M.J., Brooks T.A., Egleton R.D., Davis T.P. (2007). Peripheral inflammatory hyperalgesia modulates morphine delivery to the brain: A role for P-glycoprotein. J. Neurochem..

[57] Huber J.D., Witt K.A., Hom S., Egleton R.D., Mark K.S., Davis T.P. (2001). Inflammatory pain alters blood-brain barrier permea-bility and tight junctional protein expression. Am. J. Physiol. Heart Circ. Physiol..

[58] Campos C.R., Ocheltree S.M., Hom S., Egleton R.D., Davis T.P. (2008). Nociceptive inhibition prevents inflammatory pain induced changes in the blood–brain barrier. Brain Res..

[59] Ronaldson P.T., Demarco K.M., Sanchez-Covarrubias L., Solinsky C.M., Davis T.P. (2009). Transforming growth factor-beta sig-naling alters substrate permeability and tight junction protein expression at the blood-brain barrier during inflammatory pain. J. Cereb. Blood Flow Metab..

[60] Lampugnani M.G., Dejana E. (2007). Adherens junctions in endothelial cells regulate vessel maintenance and angiogenesis. Thromb. Res..

[61] Williams M.J., Lowrie M.B., Bennett J.P., Firth J.A., Clark P. (2005). Cadherin-10 is a novel blood–brain barrier adhesion molecule in human and mouse. Brain Res..

[62] Meng W., Takeichi M. (2009). Adherens Junction: Molecular Architecture and Regulation. Cold Spring Harb. Perspect. Biol..

[63] Artus C. (2014). The Wnt/planar cell polarity signaling pathway contributes to the integrity of tight junctions in brain endo-thelial cells. J. Cereb. Blood Flow Metab..

[64] Laksitorini M.D. (2019). Modulation of Wnt/beta-catenin signaling promotes blood-brain barrier phenotype in cultured brain endothelial cells. Sci. Rep..

[65] Steiner E., Enzmann G.U., Lyck R., Lin S., Rüegg M.A., Kroger S., Engelhardt B. (2014). The heparan sulfate proteoglycan agrin contributes to barrier properties of mouse brain endothelial cells by stabilizing adherens junctions. Cell Tissue Res..

[66] Willis C.L. (2013). Partial recovery of the damaged rat blood-brain barrier is mediated by adherens junction complexes, ex-tracellular matrix remodeling and macrophage infiltration following focal astrocyte loss. Neuroscience.

[67] Nakano-Doi A., Sakuma R., Matsuyama T., Nakagomi T. (2018). Ischemic stroke activates the VE-cadherin promoter and increases VE-cadherin expression in adult mice. Histol. Histopathol..

[68] Oldendorf W.H., Cornford M.E., Brown W.J. (1977). The large apparent work capability of the blood-brain barrier: A study of the mitochondrial content of capillary endothelial cells in brain and other tissues of the rat. Ann. Neurol..

[69] Yang J., Reilly B.G., Davis T.P., Ronaldson P.T. (2018). Modulation of Opioid Transport at the Blood-Brain Barrier by Altered ATP-Binding Cassette (ABC) Transporter Expression and Activity. Pharmaceutics.

[70] Lin L., Yee S.W., Kim R.B., Giacomini K.M. (2015). SLC transporters as therapeutic targets: Emerging opportunities. Nat. Rev. Drug Discov..

[71] Ose A., Kusuhara H., Endo C., Tohyama K., Miyajima M., Kitamura S., Sugiyama Y. (2010). Functional Characterization of Mouse Organic Anion Transporting Peptide 1a4 in the Uptake and Efflux of Drugs Across the Blood-Brain Barrier. Drug Metab. Dispos..

[72] Roberts L., Black D., Raman C., Woodford K., Zhou M., Haggerty J., Yan A., Cwirla S., Grindstaff K. (2008). Subcellular localization of transporters along the rat blood–brain barrier and blood–cerebral-spinal fluid barrier by in vivo biotinylation. Neuroscience.

[73] Gao B., Stieger B., Noé B., Fritschy J.-M., Meier P.J. (1999). Localization of the organic anion transporting polypeptide 2 (Oatp2) in capillary endothelium and choroid plexus epithelium of rat brain. J. Histochem. Cytochem..

[74] Roberts L.M., Woodford K., Zhou M., Black D.S., Haggerty J.E., Tate E.H., Grindstaff K.K., Mengesha W., Raman C., Zerangue N. (2008). Expression of the Thyroid Hormone Transporters Monocarboxylate Transporter-8 (SLC16A2) and Organic Ion Transporter-14 (SLCO1C1) at the Blood-Brain Barrier. Endocrinology.

[75] Kubo Y., Ohtsuki S., Uchida Y., Terasaki T. (2015). Quantitative Determination of Luminal and Abluminal Membrane Distributions of Transporters in Porcine Brain Capillaries by Plasma Membrane Fractionation and Quantitative Targeted Proteomics. J. Pharm. Sci..

[76] Gao B., Hagenbuch B., Kullak-Ublick G.A., Benke D., Aguzzi A., Meier P.J. (2000). Organic anion-transporting polypeptides mediate transport of opioid peptides across blood-brain barrier. J. Pharmacol. Exp. Ther..

[77] Kalliokoski A., Niemi M. (2009). Impact of OATP transporters on pharmacokinetics. J. Cereb. Blood Flow Metab..

[78] Lee W. (2005). Polymorphisms in human organic anion-transporting polypeptide 1A2 (OATP1A2): Implications for altered drug disposition and central nervous system drug entry. J. Biol. Chem..

[79] Bronger H., Koenig J., Kopplow K., Steiner H.-H., Ahmadi R., Herold-Mende C., Keppler D., Nies A.T. (2005). ABCC Drug Efflux Pumps and Organic Anion Uptake Transporters in Human Gliomas and the Blood-Tumor Barrier. Cancer Res..

[80] Sekhar G.N., Georgian A.R., Sanderson L., Vizcay-Barrena G., Brown R.C., Muresan P., Fleck R., Thomas S.A. (2017). Organic cation transporter 1 (OCT1) is involved in pentamidine transport at the human and mouse blood-brain barrier (BBB). PLoS ONE.

[81] Lin C.-J., Tai Y., Huang M.-T., Tsai Y.-F., Hsu H.-J., Tzen K.-Y., Liou H.-H. (2010). Cellular localization of the organic cation transporters, OCT1 and OCT2, in brain microvessel endothelial cells and its implication for MPTP transport across the blood-brain barrier and MPTP-induced dopaminergic toxicity in rodents. J. Neurochem..

[82] Soontornmalai A., Vlaming M.L., Fritschy J.M. (2006). Differential, strain-specific cellular and subcellular distribution of multi-drug transporters in murine choroid plexus and blood-brain barrier. Neuroscience.

[83] Beaulieu É., Demeule M., Ghitescu L., Béliveau R. (1997). P-glycoprotein is strongly expressed in the luminal membranes of the endothelium of blood vessels in the brain. Biochem. J..

[84] Bendayan R., Ronaldson P.T., Gingras D., Bendayan M. (2006). In Situ Localization of P-glycoprotein (ABCB1) in Human and Rat Brain. J. Histochem. Cytochem..

[85] Tsuji A., Terasaki T., Takabatake Y., Tenda Y., Tamai I., Yamashima T., Moritani S., Tsuruo T., Yamashita J. (1992). P-glycoprotein as the drug efflux pump in primary cultured bovine brain capillary endothelial cells. Life Sci..

[86] Watanabe D. (2021). Characterization of a Primate Blood-Brain Barrier Co-Culture Model Prepared from Primary Brain En-dothelial Cells, Pericytes and Astrocytes. Pharmaceutics.

[87] Virgintino D., Robertson D., Errede M., Benagiano V., Girolamo F., Maiorano E., Roncali L., Bertossi M. (2002). Expression of P-Glycoprotein in Human Cerebral Cortex Microvessels. J. Histochem. Cytochem..

[88] Bakhsheshian J., Wei B.-R., Hall M.D., Simpson R.M., Gottesman M.M. (2016). In Vivo Bioluminescent Imaging of ATP-Binding Cassette Transporter-Mediated Efflux at the Blood–Brain Barrier. Adv. Struct. Saf. Stud..

[89] Hori S. (2004). Functional expression of rat ABCG2 on the luminal side of brain capillaries and its enhancement by astro-cyte-derived soluble factor(s). J. Neurochem..

[90] Cooray H.C., Blackmore C.G., Maskell L., Barrand M.A. (2002). Localisation of breast cancer resistance protein in microvessel endothelium of human brain. NeuroReport.

[91] Kilic E. (2008). ABCC1: A gateway for pharmacological compounds to the ischaemic brain. Brain.

[92] Leggas M., Adachi M., Scheffer G.L., Sun D., Wielinga P., Du G., Mercer K.E., Zhuang Y., Panetta J.C., Johnston B. (2004). Mrp4 Confers Resistance to Topotecan and Protects the Brain from Chemotherapy. Mol. Cell. Biol..

[93] Gutmann H., Török M., Fricker G., Huwyler J., Beglinger C., Drewe J. (1999). Modulation of multidrug resistance protein expression in porcine brain capillary endothelial cells in vitro. Drug Metab. Dispos..

[94] Zhang Y. (2004). Plasma membrane localization of multidrug resistance-associated protein homologs in brain capillary endo-thelial cells. J. Pharmacol. Exp. Ther..

[95] Nies A., Jedlitschky G., König J., Herold-Mende C., Steiner H., Schmitt H.-P., Keppler D. (2004). Expression and immunolocalization of the multidrug resistance proteins, MRP1–MRP6 (ABCC1–ABCC6), in human brain. Neuroscience.

[96] Klaassen C.D., Aleksunes L.M. (2010). Xenobiotic, Bile Acid, and Cholesterol Transporters: Function and Regulation. Pharmacol. Rev..

[97] Grube M., Hagen P., Jedlitschky G. (2018). Neurosteroid Transport in the Brain: Role of ABC and SLC Transporters. Front. Pharmacol..

[98] Sekhar G.N., Fleckney A.L., Boyanova S.T., Rupawala H., Lo R., Wang H., Farag D.B., Rahman K.M., Broadstock M., Reeves S. (2019). Region-specific blood–brain barrier transporter changes leads to increased sensitivity to amisulpride in Alzheimer’s disease. Fluids Barriers CNS.

[99] Sandoval P.J., Zorn K.M., Clark A.M., Ekins S., Wright S.H. (2018). Assessment of Substrate-Dependent Ligand Interactions at the Organic Cation Transporter OCT2 Using Six Model Substrates. Mol. Pharmacol..

[100] Ronaldson P.T. (2021). Transport Properties of Statins by Organic Anion Transporting Polypeptide 1A2 and Regulation by Transforming Growth Factor-beta Signaling in Human Endothelial Cells. J. Pharmacol. Exp. Ther..

[101] Amin M.L. (2013). P-glycoprotein Inhibition for Optimal Drug Delivery. Drug Target Insights.

[102] Zhu Q.-Y., Tang S., Yang X.-Q., Ding H., Liu X.-D., Zeng X.-B., Huang X.-P., Deng C.-Q. (2022). Borneol enhances the protective effect against cerebral ischemia/reperfusion injury by promoting the access of astragaloside IV and the components of Panax notoginseng saponins into the brain. Phytomedicine.

[103] Chen X., Zhou Z.-W., Xue C.C., Li X.-X., Zhou S.-F. (2007). Role of P-glycoprotein in restricting the brain penetration of tanshinone IIA, a major active constituent from the root ofSalvia miltiorrhizaBunge, across the blood–brain barrier. Xenobiotica.

[104] Palmeira A., Sousa M.E., Vasconcelos M.H., Pinto M. (2012). Three Decades of P-gp Inhibitors: Skimming Through Several Generations and Scaffolds. Curr. Med. Chem..

[105] Binkhathlan Z., Lavasanifar A. (2013). P-glycoprotein inhibition as a therapeutic approach for overcoming multidrug resistance in cancer: Current status and future perspectives. Curr. Cancer Drug Targets.

[106] Kalvass J.C. (2013). Why clinical modulation of efflux transport at the human blood-brain barrier is unlikely: The ITC evi-dence-based position. Clin. Pharmacol. Ther..

[107] Polli J.W. (2009). An unexpected synergist role of P-glycoprotein and breast cancer resistance protein on the central nervous system penetration of the tyrosine kinase inhibitor lapatinib (N-{3-chloro-4-[(3-fluorobenzyl)oxy]phenyl}-6-[5-({[2-(methylsulfonyl)ethyl]amino }methyl)-2-furyl]-4-quinazolinamine; GW572016). Drug Metab. Dispos..

[108] Gomez-Zepeda D., Taghi M., Scherrmann J.-M., Decleves X., Menet M.-C. (2019). ABC Transporters at the Blood–Brain Interfaces, Their Study Models, and Drug Delivery Implications in Gliomas. Pharmaceutics.

[109] Ohtsuki S. (2013). Quantitative targeted absolute proteomic analysis of transporters, receptors and junction proteins for vali-dation of human cerebral microvascular endothelial cell line hCMEC/D3 as a human blood-brain barrier model. Mol. Pharm..

[110] Billington S., Salphati L., Hop C.E.C.A., Chu X., Evers R., Burdette D., Rowbottom C., Lai Y., Xiao G., Humphreys W.G. (2019). Interindividual and Regional Variability in Drug Transporter Abundance at the Human Blood–Brain Barrier Measured by Quantitative Targeted Proteomics. Clin. Pharmacol. Ther..

[111] Robey R.W., To K.K., Polgar O., Dohse M., Fetsch P., Dean M., Bates S.E. (2009). ABCG2: A perspective. Adv. Drug Deliv. Rev..

[112] Breedveld P., Pluim D., Cipriani G., Dahlhaus F., Van Eijndhoven M.A.J., De Wolf C.J.F., Kuil A., Beijnen J.H., Scheffer G.L., Jansen G. (2007). The Effect of Low pH on Breast Cancer Resistance Protein (ABCG2)-Mediated Transport of Methotrexate, 7-Hydroxymethotrexate, Methotrexate Diglutamate, Folic Acid, Mitoxantrone, Topotecan, and Resveratrol in In Vitro Drug Transport Models. Mol. Pharmacol..

[113] Liu L., Miao M., Chen Y., Wang Z., Sun B., Liu X. (2018). Altered Function and Expression of ABC Transporters at the Blood–Brain Barrier and Increased Brain Distribution of Phenobarbital in Acute Liver Failure Mice. Front. Pharmacol..

[114] Zamek-Gliszczynski M.J. (2011). Efflux transport is an important determinant of ethinylestradiol glucuronide and ethi-nylestradiol sulfate pharmacokinetics. Drug Metab. Dispos..

[115] Lee Y.-J., Kusuhara H., Jonker J., Schinkel A.H., Sugiyama Y. (2005). Investigation of Efflux Transport of Dehydroepiandrosterone Sulfate and Mitoxantrone at the Mouse Blood-Brain Barrier: A Minor Role of Breast Cancer Resistance Protein. J. Pharmacol. Exp. Ther..

[116] Tornabene E., Helms H.C.C., Pedersen S.F., Brodin B. (2019). Effects of oxygen-glucose deprivation (OGD) on barrier properties and mRNA transcript levels of selected marker proteins in brain endothelial cells/astrocyte co-cultures. PLoS ONE.

[117] Ronaldson P.T., Finch J.D., DeMarco K.M., Quigley C.E., Davis T.P. (2011). Inflammatory Pain Signals an Increase in Functional Expression of Organic Anion Transporting Polypeptide 1a4 at the Blood-Brain Barrier. J. Pharmacol. Exp. Ther..

[118] Ronaldson P.T., Davis T.P. (2013). Targeted Drug Delivery to Treat Pain and Cerebral Hypoxia. Pharmacol. Rev..

[119] Thompson B.J., Sanchez-Covarrubias L., Slosky L.M., Zhang Y., Laracuente M.-L., Ronaldson P.T. (2014). Hypoxia/Reoxygenation Stress Signals an Increase in Organic Anion Transporting polypeptide 1a4 (Oatp1a4) at the Blood–Brain Barrier: Relevance to CNS Drug Delivery. J. Cereb. Blood Flow Metab..

[120] Abdullahi W. (2018). Functional Expression of Organic Anion Transporting Polypeptide 1a4 Is Regulated by Transforming Growth Factor-beta/Activin Receptor-like Kinase 1 Signaling at the Blood-Brain Barrier. Mol. Pharmacol..

[121] Abdullahi W. (2017). Bone morphogenetic protein-9 increases the functional expression of organic anion transporting poly-peptide 1a4 at the blood-brain barrier via the activin receptor-like kinase-1 receptor. J. Cereb. Blood Flow Metab..

[122] Brzica H., Abdullahi W., Reilly B.G., Ronaldson P.T. (2018). Sex-specific differences in organic anion transporting polypeptide 1a4 (Oatp1a4) functional expression at the blood–brain barrier in Sprague–Dawley rats. Fluids Barriers CNS.

[123] Al-Majdoub Z.M., Al Feteisi H., Achour B., Warwood S., Neuhoff S., Rostami-Hodjegan A., Barber J. (2019). Proteomic Quantification of Human Blood–Brain Barrier SLC and ABC Transporters in Healthy Individuals and Dementia Patients. Mol. Pharm..

[124] Liu H., Yu N., Lu S., Ito S., Zhang X., Prasad B., He E., Lu X., Li Y., Wang F. (2015). Solute Carrier Family of the Organic Anion-Transporting Polypeptides 1A2– Madin-Darby Canine Kidney II: A Promising In Vitro System to Understand the Role of Organic Anion-Transporting Polypeptide 1A2 in Blood-Brain Barrier Drug Penetration. Drug Metab. Dispos..

[125] Albekairi T.H., Vaidya B., Patel R., Nozohouri S., Villalba H., Zhang Y., Lee Y.S., Al-Ahmad A., Abbruscato T.J. (2019). Brain Delivery of a Potent Opioid Receptor Agonist, Biphalin during Ischemic Stroke: Role of Organic Anion Transporting Polypeptide (OATP). Pharmaceutics.

[126] Yang L., Islam M.R., Karamyan V.T., Abbruscato T.J. (2015). In vitro and in vivo efficacy of a potent opioid receptor agonist, biphalin, compared to subtype-selective opioid receptor agonists for stroke treatment. Brain Res..

[127] Yang L., Shah K., Wang H., Karamyan V.T., Abbruscato T.J. (2011). Characterization of Neuroprotective Effects of Biphalin, an Opioid Receptor Agonist, in a Model of Focal Brain Ischemia. J. Pharmacol. Exp. Ther..

[128] Yang L., Wang H., Shah K., Karamyan V.T., Abbruscato T.J. (2011). Opioid receptor agonists reduce brain edema in stroke. Brain Res..

[129] Islam M.R., Yang L., Lee Y.S., Hruby V.J., Karamyan V.T., Abbruscato T.J. (2016). Enkephalin-Fentanyl Multifunctional Opioids as Potential Neuroprotectants for Ischemic Stroke Treatment. Curr. Pharm. Des..

[130] Fang S., Xu H., Lu J., Zhu Y., Jiang H. (2013). Neuroprotection by the Kappa-Opioid Receptor Agonist, BRL52537, is Mediated via Up-Regulating Phosphorylated Signal Transducer and Activator of Transcription-3 in Cerebral Ischemia/Reperfusion Injury in Rats. Neurochem. Res..

[131] Eftekhar-Vaghefi S., Esmaeili-Mahani S., Elyasi L., Abbasnejad M. (2015). Involvement of Mu Opioid Receptor Signaling in the Protective Effect of Opioid against 6-Hydroxydopamine-Induced SH-SY5Y Human Neuroblastoma Cells Apoptosis. Basic Clin. Neurosci. J..

[132] Sung J.-H., Yu K.-H., Park J.-S., Tsuruo T., Kim D.-D., Shim C.-K., Chung S.-J. (2005). Saturable distribution of tacrine into the striatal extracellular fluid of the rat: Evidence of involvement of multiple organic cation transporters in the transport. Drug Metab. Dispos..

[133] Wu K.-C., Lu Y.-H., Peng Y.-H., Tsai T.-F., Kao Y.-H., Yang H.-T., Lin C.-J. (2014). Decreased Expression of Organic Cation Transporters, Oct1 and Oct2, in Brain Microvessels and its Implication to MPTP-Induced Dopaminergic Toxicity in Aged Mice. J. Cereb. Blood Flow Metab..

[134] Betterton R.D., Davis T.P., Ronaldson P.T. (2021). Organic Cation Transporter (OCT/OCTN) Expression at Brain Barrier Sites: Focus on CNS Drug Delivery. Handb. Exp. Pharmacol..

[135] Hiasa M., Matsumoto T., Komatsu T., Moriyama Y. (2006). Wide variety of locations for rodent MATE1, a transporter protein that mediates the final excretion step for toxic organic cations. Am. J. Physiol. Physiol..

[136] Chaves C., Campanelli F., Chapy H., Gomez-Zepeda D., Glacial F., Smirnova M., Taghi M., Pallud J., Perrière N., Declèves X. (2020). An Interspecies Molecular and Functional Study of Organic Cation Transporters at the Blood-Brain Barrier: From Rodents to Humans. Pharmaceutics.

[137] Geier E.G., Chen E.C., Webb A., Papp A.C., Yee S.W., Sadee W., Giacomini K.M. (2013). Profiling Solute Carrier Transporters in the Human Blood–Brain Barrier. Clin. Pharmacol. Ther..

[138] Mehta D.C., Short J.L., Nicolazzo J.A. (2013). Memantine Transport across the Mouse Blood–Brain Barrier Is Mediated by a Cationic Influx H+ Antiporter. Mol. Pharm..

[139] Busch A.E., Karbach U., Miska D., Gorboulev V., Akhoundova A., Volk C., Arndt P., Ulzheimer J.C., Sonders M.S., Baumann C. (1998). Human Neurons Express the Polyspecific Cation Transporter hOCT2, Which Translocates Monoamine Neurotransmitters, Amantadine, and Memantine. Mol. Pharmacol..

[140] Graham G.G., Punt J., Arora M., Day R.O., Doogue M.P., Duong J.K., Furlong T.J., Greenfield J.R., Greenup L.C., Kirkpatrick C. (2011). Clinical Pharmacokinetics of Metformin. Clin. Pharmacokinet..

[141] Wrobel M.P. (2017). Metformin—A new old drug. Endokrynol. Pol..

[142] Sharma S., Nozohouri S., Vaidya B., Abbruscato T. (2021). Repurposing metformin to treat age-related neurodegenerative disorders and ischemic stroke. Life Sci..

[143] Chen Y. (2009). Antidiabetic drug metformin (GlucophageR) increases biogenesis of Alzheimer’s amyloid peptides via up-regulating BACE1 transcription. Proc. Natl. Acad. Sci. USA.

[144] Platt S.R., Holmes S.P., Howerth E.W., Duberstein K.J.J., Dove C.R., Kinder H.A., Wyatt E.L., Linville A.V., Lau V.W., Stice S.L. (2014). Development and characterization of a Yucatan miniature biomedical pig permanent middle cerebral artery occlusion stroke model. Exp. Transl. Stroke Med..

[145] Cook D.J., Tymianski M. (2012). Nonhuman Primate Models of Stroke for Translational Neuroprotection Research. Neurotherapeutics.

[146] Narayan S.K., Cherian S.G., Phaniti P.B., Chidambaram S.B., Vasanthi A.H.R., Arumugam M. (2021). Preclinical animal studies in ischemic stroke: Challenges and some solutions. Anim. Model. Exp. Med..

[147] Debatisse J., Wateau O., Cho T.-H., Costes N., Merida I., Léon C., Langlois J.-B., Taborik F., Verset M., Portier K. (2021). A non-human primate model of stroke reproducing endovascular thrombectomy and allowing long-term imaging and neurological read-outs. J. Cereb. Blood Flow Metab..

[148] Kumar N., Lee J.J., Perlmutter J.S., Derdeyn C.P. (2009). Cervical Carotid and Circle of Willis Arterial Anatomy of Macaque Monkeys: A Comparative Anatomy Study. Anat. Rec..

[149] Kassel N.F., Langfitt T.W. (1965). Variations in the circle of Willis in Macaca mulatta. Anat. Rec..

[150] Hoshi Y., Uchida Y., Tachikawa M., Inoue T., Ohtsuki S., Terasaki T. (2013). Quantitative Atlas of Blood–Brain Barrier Transporters, Receptors, and Tight Junction Proteins in Rats and Common Marmoset. J. Pharm. Sci..

[151] Auvity S., Caillé F., Marie S., Wimberley C., Bauer M., Langer O., Buvat I., Goutal S., Tournier N. (2018). P-Glycoprotein (ABCB1) Inhibits the Influx and Increases the Efflux of 11C-Metoclopramide Across the Blood–Brain Barrier: A PET Study on Nonhuman Primates. J. Nucl. Med..

[152] García-Varela L., Arif W.M., García D.V., Kakiuchi T., Ohba H., Harada N., Tago T., Elsinga P.H., Tsukada H., Colabufo N.A. (2020). Pharmacokinetic Modeling of [18F]MC225 for Quantification of the P-Glycoprotein Function at the Blood–Brain Barrier in Non-Human Primates with PET. Mol. Pharm..

[153] Zhang K., Sejnowski T.J. (2000). A universal scaling law between gray matter and white matter of cerebral cortex. Proc. Natl. Acad. Sci. USA.

[154] Watanabe H. (2001). MR-based statistical atlas of the Gottingen minipig brain. Neuroimage.

[155] Sozmen E.G., Hinman J.D., Carmichael S.T. (2012). Models That Matter: White Matter Stroke Models. Neurotherapeutics.

[156] Okuyama S., Okuyama J., Tamatsu Y., Shimada K., Hoshi H., Iwai J. (2004). The arterial circle of Willis of the mouse helps to decipher secrets of cerebral vascular accidents in the human. Med. Hypotheses.

[157] McColl B.W., Carswell H.V., McCulloch J., Horsburgh K. (2004). Extension of cerebral hypoperfusion and ischaemic pathology beyond MCA territory after intraluminal filament occlusion in C57Bl/6J mice. Brain Res..

[158] Uchida Y., Yagi Y., Takao M., Tano M., Umetsu M., Hirano S., Usui T., Tachikawa M., Terasaki T. (2020). Comparison of Absolute Protein Abundances of Transporters and Receptors among Blood–Brain Barriers at Different Cerebral Regions and the Blood–Spinal Cord Barrier in Humans and Rats. Mol. Pharm..

[159] Longa E.Z., Weinstein P.R., Carlson S., Cummins R. (1989). Reversible middle cerebral artery occlusion without craniectomy in rats. Stroke.

[160] Belayev L., Alonso O.F., Busto R., Zhao W., Ginsberg M.D. (1996). Middle Cerebral Artery Occlusion in the Rat by Intraluminal Suture. Stroke.

[161] Barthels D., Das H. (2020). Current advances in ischemic stroke research and therapies. Biochim. Biophys. Acta (BBA) Mol. Basis Dis..

[162] Vital S.A., Gavins F.N.E. (2016). Surgical Approach for Middle Cerebral Artery Occlusion and Reperfusion Induced Stroke in Mice. J. Vis. Exp..

[163] Lam T.I., Wise P.M., O’Donnell M.E. (2009). Cerebral microvascular endothelial cell Na/H exchange: Evidence for the presence of NHE1 and NHE2 isoforms and regulation by arginine vasopressin. Am. J. Physiol. Cell Physiol..

[164] Wallace B.K., Foroutan S., O’Donnell M.E. (2011). Ischemia-induced stimulation of Na-K-Cl cotransport in cerebral microvas-cular endothelial cells involves AMP kinase. Am. J. Physiol. Cell Physiol..

[165] Wallace B.K., Jelks K.A., O’Donnell M.E. (2012). Ischemia-induced stimulation of cerebral microvascular endothelial cell Na-K-Cl cotransport involves p38 and JNK MAP kinases. Am. J. Physiol. Cell Physiol..

[166] Zhang F.H. (2013). Rosiglitazone attenuates hyperglycemia-enhanced hemorrhagic transformation after transient focal is-chemia in rats. Neuroscience.

[167] Ismael S., Nasoohi S., Yoo A., Ahmed H.A., Ishrat T. (2020). Tissue Plasminogen Activator Promotes TXNIP-NLRP3 Inflammasome Activation after Hyperglycemic Stroke in Mice. Mol. Neurobiol..

[168] Steele E.C., Guo Q., Namura S. (2008). Filamentous Middle Cerebral Artery Occlusion Causes Ischemic Damage to the Retina in Mice. Stroke.

[169] Sommer C.J. (2017). Ischemic stroke: Experimental models and reality. Acta Neuropathol..

[170] Wu D., Chen J., Wang B., Zhang M., Shi J., Ma Y., Zhu Z., Yan F., He X., Li S. (2016). Endovascular ischemic stroke models of adult rhesus monkeys: A comparison of two endovascular methods. Sci. Rep..

[171] Overgaard K., Sereghy T., Boysen G., Pedersen H., Høyer S., Diemer N.H. (1992). A Rat Model of Reproducible Cerebral Infarction Using Thrombotic Blood Clot Emboli. J. Cereb. Blood Flow Metab..

[172] Busch E., Krüger K., Hossmann K.-A. (1997). Improved model of thromboembolic stroke and rt-PA induced reperfusion in the rat. Brain Res..

[173] Zhang Z., Chopp M., Zhang R.L., Goussev A. (1997). A Mouse Model of Embolic Focal Cerebral Ischemia. J. Cereb. Blood Flow Metab..

[174] Zhang R.L., Chopp M., Zhang Z.G., Jiang Q., Ewing J.R. (1997). A rat model of focal embolic cerebral ischemia. Brain Res..

[175] Chen Y., Zhu W., Zhang W., Libal N., Murphy S.J., Offner H., Alkayed N.J. (2015). A novel mouse model of thromboembolic stroke. J. Neurosci. Methods.

[176] Ren M., Lin Z.-J., Qian H., Choudhury G.R., Liu R., Liu H., Yang S.-H. (2012). Embolic middle cerebral artery occlusion model using thrombin and fibrinogen composed clots in rat. J. Neurosci. Methods.

[177] Carmichael S.T. (2005). Rodent models of focal stroke: Size, mechanism, and purpose. NeuroRX.

[178] Busch E., Krüger K., Fritze K., Allegrini P.R., Hoehn-Berlage M., Hossmann K.A. (1997). Blood-Brain Barrier Disturbances After rt-PA Treatment of Thromboembolic Stroke in the Rat. Acta Neurochir. Suppl..

[179] Kleinschnitz C., Fluri F., Schuhmann M. (2015). Animal models of ischemic stroke and their application in clinical research. Drug Des. Dev. Ther..

[180] Lu H., Li Y., Yuan L., Li H., Lu X., Tong S. (2014). Induction and imaging of photothrombotic stroke in conscious and freely moving rats. J. Biomed. Opt..

[181] Yu C.-L., Zhou H., Chai A.-P., Yang Y.-X., Mao R.-R., Xu L. (2015). Whole-scale neurobehavioral assessments of photothrombotic ischemia in freely moving mice. J. Neurosci. Methods.

[182] Kleinschnitz C., Braeuninger S., Pham M., Austinat M., Nölte I., Renné T., Nieswandt B., Bendszus M., Stoll G. (2008). Blocking of Platelets or Intrinsic Coagulation Pathway–Driven Thrombosis Does Not Prevent Cerebral Infarctions Induced by Photothrombosis. Stroke.

[183] Frederix K., Chauhan A.K., Kisucka J., Zhao B.-Q., Hoff E.I., Spronk H.M.H., Cate H.T., Wagner D.D. (2007). Platelet adhesion receptors do not modulate infarct volume after a photochemically induced stroke in mice. Brain Res..

[184] Jiang W. (2006). Establishing a photothrombotic ’ring’ stroke model in adult mice with late spontaneous reperfusion: Quanti-tative measurements of cerebral blood flow and cerebral protein synthesis. J. Cereb. Blood Flow Metab..

[185] Wester P., Watson B.D., Prado R., Dietrich W.D. (1995). A Photothrombotic ‘Ring’ Model of Rat Stroke-in-Evolution Displaying Putative Penumbral Inversion. Stroke.

[186] Hu X., Wester P., Brännström T., Watson B.D., Gu W. (2001). Progressive and reproducible focal cortical ischemia with or without late spontaneous reperfusion generated by a ring-shaped, laser-driven photothrombotic lesion in rats. Brain Res. Protoc..

[187] Virley D., Hadingham S.J., Roberts J.C., Farnfield B., Elliott H., Whelan G., Golder J., David C., Parsons A.A., Hunter A.J. (2004). A New Primate Model of Focal Stroke: Endothelin-1—Induced Middle Cerebral Artery Occlusion and Reperfusion in the Common Marmoset. J. Cereb. Blood Flow Metab..

[188] Haynes W., Webb D.J. (1998). Endothelin as a regulator of cardiovascular function in health and disease. J. Hypertens..

[189] Abeysinghe H.C.S., Roulston C.L. (2018). A Complete Guide to Using the Endothelin-1 Model of Stroke in Conscious Rats for Acute and Long-Term Recovery Studies. Program. Necrosis.

[190] Ansari S., Azari H., Caldwell K.J., Regenhardt R.W., Hedna V.S., Waters M.F., Hoh B.L., Mecca A.P. (2013). Endothelin-1 Induced Middle Cerebral Artery Occlusion Model for Ischemic Stroke with Laser Doppler Flowmetry Guidance in Rat. J. Vis. Exp..

[191] Sharkey J., Ritchie I.M., Kelly P.A. (1993). Perivascular microapplication of endothelin-1: A new model of focal cerebral ischaemia in the rat. J. Cereb. Blood Flow Metab..

[192] Biernaskie J., Corbett D., Peeling J., Wells J., Lei H. (2001). A serial MR study of cerebral blood flow changes and lesion development following endothelin-1-induced ischemia in rats. Magn. Reson. Med..

[193] Bauer B., Hartz A.M.S., Miller D.S. (2007). Tumor Necrosis Factor α and Endothelin-1 Increase P-Glycoprotein Expression and Transport Activity at the Blood-Brain Barrier. Mol. Pharmacol..

[194] Hartz A.M.S., Bauer B., Fricker G., Miller D.S. (2004). Rapid Regulation of P-Glycoprotein at the Blood-Brain Barrier by Endothelin-1. Mol. Pharmacol..

[195] Harati R., Villégier A.-S., Banks W.A., Mabondzo A. (2012). Susceptibility of juvenile and adult blood–brain barrier to endothelin-1: Regulation of P-glycoprotein and breast cancer resistance protein expression and transport activity. J. Neuroinflamm..

[196] Chen R., Ovbiagele B., Feng W. (2016). Diabetes and Stroke: Epidemiology, Pathophysiology, Pharmaceuticals and Outcomes. Am. J. Med. Sci..

[197] Putaala J., Liebkind R., Gordin D., Thorn L.M., Haapaniemi E., Forsblom C., Groop P.-H., Kaste M., Tatlisumak T. (2011). Diabetes mellitus and ischemic stroke in the young: Clinical features and long-term prognosis. Neurology.

[198] Khoury J.C. (2013). Diabetes mellitus: A risk factor for ischemic stroke in a large biracial population. Stroke.

[199] Lee M., Saver J., Hong K.-S., Song S., Chang K.-H., Ovbiagele B. (2012). Effect of pre-diabetes on future risk of stroke: Meta-analysis. BMJ.

[200] Huber J.D. (2008). Diabetes, Cognitive Function, and the Blood-Brain Barrier. Curr. Pharm. Des..

[201] Qiao J., Lawson C., Rentrup K.F.G., Kulkarni P., Ferris C.F. (2020). Evaluating blood–brain barrier permeability in a rat model of type 2 diabetes. J. Transl. Med..

[202] Hawkins B.T., Lundeen T.F., Norwood K.M., Brooks H.L., Egleton R.D. (2006). Increased blood–brain barrier permeability and altered tight junctions in experimental diabetes in the rat: Contribution of hyperglycaemia and matrix metalloproteinases. Diabetologia.

[203] Banks W.A. (2020). The Blood-Brain Barrier Interface in Diabetes Mellitus: Dysfunctions, Mechanisms and Approaches to Treatment. Curr. Pharm. Des..

[204] Salameh T.S., Mortell W.G., Logsdon A.F., Butterfield D.A., Banks W.A. (2019). Disruption of the hippocampal and hypothalamic blood–brain barrier in a diet-induced obese model of type II diabetes: Prevention and treatment by the mitochondrial carbonic anhydrase inhibitor, topiramate. Fluids Barriers CNS.

[205] Prasad S. (2014). Diabetes Mellitus and Blood-Brain Barrier Dysfunction: An Overview. J. Pharmacovigil..

[206] Van Sloten T.T. (2020). Cerebral microvascular complications of type 2 diabetes: Stroke, cognitive dysfunction, and depression. Lancet Diabetes Endocrinol..

[207] Mergenthaler P., Lindauer U., Dienel G.A., Meisel A. (2013). Sugar for the brain: The role of glucose in physiological and pathological brain function. Trends Neurosci..

[208] Cornford E.M., Hyman S., Pardridge W.M. (1993). An Electron Microscopic Immunogold Analysis of Developmental Up-Regulation of the Blood—Brain Barrier GLUT1 Glucose Transporter. J. Cereb. Blood Flow Metab..

[209] Farrell C.L., Pardridge W.M. (1991). Blood-brain barrier glucose transporter is asymmetrically distributed on brain capillary endothelial lumenal and ablumenal membranes: An electron microscopic immunogold study. Proc. Natl. Acad. Sci. USA.

[210] Gerhart D.Z., Levasseur R.J., Broderius M.A., Drewes L.R. (1989). Glucose transporter localization in brain using light and electron immunocytochemistry. J. Neurosci. Res..

[211] Simpson I.A., Vannucci S.J., DeJoseph M.R., Hawkins R.A. (2001). Glucose Transporter Asymmetries in the Bovine Blood-Brain Barrier. J. Biol. Chem..

[212] Sogin D.C., Hinkle P.C. (1980). Immunological identification of the human erythrocyte glucose transporter. Proc. Natl. Acad. Sci. USA.

[213] McAllister M.S., Krizanac-Bengez L., Macchia F., Naftalin R., Pedley K.C., Mayberg M., Marroni M., Leaman S., Stanness K.A., Janigro D. (2001). Mechanisms of glucose transport at the blood-brain barrier: An in vitro study. Brain Res..

[214] Duelli R., Maurer M., Staudt R., Heiland S., Duembgen L., Kuschinsky W. (2000). Increased cerebral glucose utilization and decreased glucose transporter Glut1 during chronic hyperglycemia in rat brain. Brain Res..

[215] Lee W.H., Bondy C.A. (1993). Ischemic injury induces brain glucose transporter gene expression. Endocrinology.

[216] Urabe T., Hattori N., Nagamatsu S., Sawa H., Mizuno Y. (2002). Expression of Glucose Transporters in Rat Brain Following Transient Focal Ischemic Injury. J. Neurochem..

[217] Gerhart D.Z., Leino R.L., Taylor W.E., Borson N.D., Drewes L.R. (1994). GLUT1 and GLUT3 gene expression in gerbil brain following brief ischemia: An in situ hybridization study. Mol. Brain Res..

[218] McCall A.L., Van Bueren A.M., Nipper V., Moholt-Siebert M., Downes H., Lessov N. (1996). Forebrain Ischemia Increases Glut1 Protein in Brain Microvessels and Parenchyma. J. Cereb. Blood Flow Metab..

[219] Zhang W.-W., Zhang L., Hou W.-K., Xu Y.-X., Xu H., Lou F.-C., Zhang Y., Wang Q. (2009). Dynamic expression of glucose transporters 1 and 3 in the brain of diabetic rats with cerebral ischemia reperfusion. Chin. Med. J..

[220] Liu H., Liu X., Jia L., Liu Y., Yang H., Wang G., Xie L. (2008). Insulin therapy restores impaired function and expression of P-glycoprotein in blood–brain barrier of experimental diabetes. Biochem. Pharmacol..

[221] Maeng H.J. (2007). Functional induction of P-glycoprotein in the blood-brain barrier of streptozotocin-induced diabetic rats: Evidence for the involvement of nuclear factor-kappaB, a nitrosative stress-sensitive transcription factor, in the regulation. Drug Metab. Dispos..

[222] Wu K.-C., Pan H.-J., Yin H.-S., Chen M.-R., Lu S.-C., Lin C.-J., Chun-Jung L. (2009). Change in P-glycoprotein and caveolin protein expression in brain striatum capillaries in New Zealand Obese mice with type 2 diabetes. Life Sci..

[223] Cornelius M.E., Wang T.W., Jamal A., Loretan C.G., Neff L.J. (2020). Tobacco Product Use Among Adults—United States, 2019. MMWR Morb. Mortal. Wkly. Rep..

[224] Feigin V.L., Roth G.A., Naghavi M., Parmar P., Krishnamurthi R., Chugh S., Mensah G.A., Norrving B., Shiue I., Ng M. (2016). Global burden of stroke and risk factors in 188 countries, during 1990–2013: A systematic analysis for the Global Burden of Disease Study. Lancet Neurol..

[225] Markidan J., Cole J.W., Cronin C.A., Merino J.G., Phipps M., Wozniak M.A., Kittner S.J. (2018). Smoking and Risk of Ischemic Stroke in Young Men. Stroke.

[226] Thrift A.G., Thayabaranathan T., Howard G., Howard V.J., Rothwell P.M., Feigin V.L., Norrving B., Donnan G.A., Cadilhac D. (2017). Global stroke statistics. Int. J. Stroke.

[227] Wolf A.P., D’Agostino R.B., Kannel W.B., Bonita R., Belanger A.J. (1988). Cigarette smoking as a risk factor for stroke. The Framingham Study. JAMA J. Am. Med. Assoc..

[228] Pan B. (2019). The relationship between smoking and stroke: A meta-analysis. Medicine.

[229] Fetterman J.L., Weisbrod R.M., Feng B., Bastin R., Tuttle S.T., Holbrook M., Baker G., Robertson R.M., Conklin D.J., Bhatnagar A. (2018). Flavorings in Tobacco Products Induce Endothelial Cell Dysfunction. Arter. Thromb. Vasc. Biol..

[230] Malek A.M. (2015). Secondhand Smoke Exposure and Stroke: The Reasons for Geographic and Racial Differences in Stroke (REGARDS) Study. Am. J. Prev. Med..

[231] Pistilli M., Howard V.J., Safford M.M., Lee B.K., Lovasi G.S., Cushman M., Malek A.M., McClure L.A., REGARDS Investigators (2019). Association of secondhand tobacco smoke exposure during childhood on adult cardiovascular disease risk among never-smokers. Ann. Epidemiol..

[232] Howard G., Burke G.L., Szklo M., Tell G.S., Eckfeldt J., Evans G., Heiss G. (1994). Active and passive smoking are associated with increased carotid wall thickness. The Atherosclerosis Risk in Communities Study. Arch. Intern. Med..

[233] Shah R.S., Cole J.W. (2010). Smoking and stroke: The more you smoke the more you stroke. Expert Rev. Cardiovasc. Ther..

[234] Penn A., Snyder C.A. (1996). 1,3 Butadiene, a Vapor Phase Component of Environmental Tobacco Smoke, Accelerates Arteriosclerotic Plaque Development. Circulation.

[235] Seo S.-B., Choe E.S., Kim K.-S., Shim S.-M. (2017). The effect of tobacco smoke exposure on the generation of reactive oxygen species and cellular membrane damage using co-culture model of blood brain barrier with astrocytes. Toxicol. Ind. Health.

[236] Abbruscato T.J., Lopez S.P., Mark K.S., Hawkins B.T., Davis T.P. (2002). Nicotine and Cotinine Modulate Cerebral Microvascular Permeability and Protein Expression of ZO-1 through Nicotinic Acetylcholine Receptors Expressed on Brain Endothelial Cells. J. Pharm. Sci..

[237] Hawkins B., Abbruscato T.J., Egleton R.D., Brown R.C., Huber J.D., Campos C.R., Davis T. (2004). Nicotine increases in vivo blood–brain barrier permeability and alters cerebral microvascular tight junction protein distribution. Brain Res..

[238] Kadry H., Noorani B., Bickel U., Abbruscato T.J., Cucullo L. (2021). Comparative assessment of in vitro BBB tight junction integrity following exposure to cigarette smoke and e-cigarette vapor: A quantitative evaluation of the protective effects of metformin using small-molecular-weight paracellular markers. Fluids Barriers CNS.

[239] Mazzone P., Tierney W., Hossain M., Puvenna V., Janigro D., Cucullo L. (2010). Pathophysiological Impact of Cigarette Smoke Exposure on the Cerebrovascular System with a Focus on the Blood-brain Barrier: Expanding the Awareness of Smoking Toxicity in an Underappreciated Area. Int. J. Environ. Res. Public Health.

[240] Pimentel E., Sivalingam K., Doke M., Samikkannu T. (2020). Effects of Drugs of Abuse on the Blood-Brain Barrier: A Brief Overview. Front. Neurosci..

[241] Abbruscato T.J., Lopez S.P., Roder K., Paulson J.R. (2004). Regulation of Blood-Brain Barrier Na,K,2Cl-Cotransporter through Phosphorylation during in Vitro Stroke Conditions and Nicotine Exposure. J. Pharmacol. Exp. Ther..

[242] Manda V.K., Mittapalli R.K., Bohn K.A., Adkins C.E., Lockman P.R. (2010). Nicotine and cotinine increases the brain penetration of saquinavir in rat. J. Neurochem..

[243] Tsao C.W., Aday A.W., Almarzooq Z.I., Alonso A., Beaton A.Z., Bittencourt M.S., Boehme A.K., Buxton A.E., Carson A.P., Commodore-Mensah Y. (2020). Heart Disease and Stroke Statistics—2022 Update: A Report From the American Heart Association. Circulation.

[244] Boyle P.M., Del Álamo J.C., Akoum N. (2021). Fibrosis, atrial fibrillation and stroke: Clinical updates and emerging mechanistic models. Heart.

[245] Hayden D.T. (2015). Rates and Determinants of 5-Year Outcomes After Atrial Fibrillation-Related Stroke: A Population Study. Stroke.

[246] Voukalis C., Shantsila E., Lip G.Y. (2017). Clinical Stroke prevention in atrial fibrillation. J. R. Coll. Physicians Edinb..

[247] Junejo R.T., Braz I.D., Lucas S., Van Lieshout J.J., Phillips A.A., Lip G.Y., Fisher J.P. (2020). Neurovascular coupling and cerebral autoregulation in atrial fibrillation. J. Cereb. Blood Flow Metab..

[248] Girouard H., Iadecola C. (2006). Neurovascular coupling in the normal brain and in hypertension, stroke, and Alzheimer disease. J. Appl. Physiol..

[249] Gardarsdottir M., Sigurdsson S., Aspelund T., Rokita H., Launer L.J., Gudnason V., Arnar D.O. (2018). Atrial fibrillation is associated with decreased total cerebral blood flow and brain perfusion. Europace.

[250] Petersen P., Kastrup J., Videbæk R., Boysen G. (1989). Cerebral Blood Flow before and after Cardioversion of Atrial Fibrillation. J. Cereb. Blood Flow Metab..

[251] Totaro R., Corridoni C., Marini C., Marsili R., Prencipe M. (1993). Transcranial Doppler evaluation of cerebral blood flow in patients with paroxysmal atrial fibrillation. Neurol. Sci..

[252] Aryal R., Patabendige A. (2021). Blood–brain barrier disruption in atrial fibrillation: A potential contributor to the increased risk of dementia and worsening of stroke outcomes?. Open Biol..

[253] Soga Y., Aiyagari G.P.V. (2011). Clinical hypertension and vascular diseases. Hypertension and Stroke.

[254] Ostwald S.K., Wasserman J., Davis S. (2006). Medications, Comorbidities, and Medical Complications in Stroke Survivors: The CAReS Study. Rehabil. Nurs..

[255] Brown D.W. (2006). Preeclampsia and the risk of ischemic stroke among young women: Results from the Stroke Prevention in Young Women Study. Stroke.

[256] Cipolla M.J., Liebeskind D.S., Chan S.-L. (2018). The importance of comorbidities in ischemic stroke: Impact of hypertension on the cerebral circulation. J. Cereb. Blood Flow Metab..

[257] Humphrey J.D. (2008). Mechanisms of arterial remodeling in hypertension: Coupled roles of wall shear and intramural stress. Hypertension.

[258] Mohammadi M.T., Dehghani G.A. (2014). Acute hypertension induces brain injury and blood–brain barrier disruption through reduction of claudins mRNA expression in rat. Pathol. Res. Pract..

[259] Nakagawa S., Ohara H., Niwa M., Yamagata K., Nibika T. (2022). Defective Function of the Blood-Brain Barrier in a Stroke-Prone Spontaneously Hypertensive Rat: Eval-uation in an In Vitro Cell Culture Model. Cell Mol. Neurobiol..

[260] Hom S., Fleegal M.A., Egleton R.D., Campos C.R., Hawkins B.T., Davis T.P. (2007). Comparative changes in the blood-brain barrier and cerebral infarction of SHR and WKY rats. Am. J. Physiol. Integr. Comp. Physiol..

[261] Yousufuddin M., Young N. (2019). Aging and ischemic stroke. Aging.

[262] Yousufuddin M., Bartley A.C., Alsawas M., Sheely H.L., Shultz J., Takahashi P.Y., Young N.P., Murad M.H. (2017). Impact of Multiple Chronic Conditions in Patients Hospitalized with Stroke and Transient Ischemic Attack. J. Stroke Cerebrovasc. Dis..

[263] Suenaga J., Hu X., Pu H., Shi Y., Hassan S.H., Xu M., Leak R., Stetler R.A., Gao Y., Chen J. (2015). White matter injury and microglia/macrophage polarization are strongly linked with age-related long-term deficits in neurological function after stroke. Exp. Neurol..

[264] Erdő F., Denes L., de Lange E. (2017). Age-associated physiological and pathological changes at the blood–brain barrier: A review. J. Cereb. Blood Flow Metab. Off. J. Int. Soc. Cereb. Blood Flow Metab..

[265] Cai W., Zhang K., Li P., Zhu L., Xu J., Yang B., Hu X., Lu Z., Chen J. (2017). Dysfunction of the neurovascular unit in ischemic stroke and neurodegenerative diseases: An aging effect. Ageing Res. Rev..

[266] Kelly K.A., Li X., Tan Z., VanGilder R.L., Rosen C.L., Huber J.D. (2009). NOX2 inhibition with apocynin worsens stroke outcome in aged rats. Brain Res..

[267] Lähteenvuo J., Rosenzweig A. (2012). Effects of aging on angiogenesis. Circ. Res..

[268] Marín C., Yubero-Serrano E.M., López-Miranda J., Perez-Jimenez F. (2013). Endothelial Aging Associated with Oxidative Stress Can Be Modulated by a Healthy Mediterranean Diet. Int. J. Mol. Sci..

[269] Zlokovic B.V. (2011). Neurovascular pathways to neurodegeneration in Alzheimer’s disease and other disorders. Nat. Rev. Neurosci..

[270] Wu B., Ueno M., Onodera M., Kusaka T., Huang C.-L., Hosomi N., Kanenishi K., Sakamoto H. (2009). Age-related changes in P-glycoprotein expression in senescence-accelerated mouse. Curr. Aging Sci..

